# Identification and histological validation of autophagy-related core genes ADRB2 and PLK2 in keloids, with integrated immune infiltration analysis

**DOI:** 10.3389/fimmu.2026.1724230

**Published:** 2026-02-09

**Authors:** Junjie Jin, Yue Jin, Bo Lu, Zhehu Jin

**Affiliations:** 1 Department of Dermatology, Yanbian University Hospital, Yanbian University Medical College, Yanji, China; 2 Keloid Research Center, Department of Dermatology, Yanbian University Medical College, Yanji, China

**Keywords:** ADRB2, autophagy, immune microenvironment, keloid, PLK2

## Abstract

**Introduction:**

Keloids are pathological fibroproliferative scars characterized by excessive collagen deposition and a lack of effective targeted therapies. Autophagy dysregulation has been linked to keloid pathogenesis, but the underlying molecular mechanisms remain unclear.

**Methods:**

Transcriptomic datasets were integrated and analyzed using differential expression analysis and weighted gene co-expression network analysis. Three machine learning algorithms—least absolute shrinkage and selection operator (LASSO), support vector machine–recursive feature elimination (SVM-RFE), and random forest—were applied to identify autophagy-related hub gene candidates in keloids. Immune infiltration and functional analyses were conducted to explore immune microenvironment alterations. Histological staining (H&E and Masson), immunohistochemistry, and Western blotting were used for tissue-level validation, while cellular experiments were performed in keloid fibroblasts with autophagy modulation.

**Results:**

ADRB2 and PLK2 were consistently identified as key autophagy-related candidate genes. Immune-related analyses suggested that these genes may be involved in remodeling the keloid immune microenvironment by influencing the abundance and functional status of multiple immune cell subsets. Histological and protein-level assays demonstrated significantly higher expression of ADRB2 and PLK2 in keloid tissues compared with adjacent normal skin. In keloid fibroblasts, fibrotic markers (COL1/COL3) and autophagy-related markers (LC3-II/LC3-I and p62) were upregulated concomitantly with ADRB2 and PLK2 at baseline. Autophagy modulation altered ADRB2 expression (decreased with EBSS and increased with chloroquine), whereas PLK2 expression remained largely unchanged.

**Discussion:**

These findings identify ADRB2 and PLK2 as under-recognized autophagy- andimmunity-related candidate biomarkers in keloids, highlighting their potential relevance asdiagnostic indicators and future therapeutic research targets.

## 1 Introduction

Keloids are chronic fibroproliferative skin disorders characterized by the persistent overgrowth of dermal connective tissue following cutaneous injury. Unlike normal scars, keloid lesions frequently extend beyond the original wound margin and rarely undergo spontaneous regression (1). Clinically, patients often experience pruritus, pain, and visible disfigurement, with a higher prevalence observed in adolescents and individuals with darker skin tones, such as those of Asian and African descent (2). In severe cases, keloids can lead to psychological distress and significant social impairment. Current evidence suggests that keloid formation is closely associated with sustained activation of dermal fibroblasts, excessive deposition of type I and type III collagen, and dysregulation across multiple wound-healing pathways (3). Key signaling cascades involved in this process include the TGF-β/Smad, Wnt/β-catenin, PI3K/Akt, and mTOR pathways (4). Moreover, immune microenvironment remodeling, fibroblast subpopulation heterogeneity, and mechanotransduction have also emerged as critical factors contributing to keloid pathophysiology (5, 6).

The integration of high-throughput omics technologies with machine learning has accelerated the discovery of pathogenic mechanisms and candidate biomarkers. For instance, studies employing bulk RNA sequencing and weighted gene co-expression network analysis (WGCNA) have identified genes associated with immune remodeling and recurrence risk, demonstrating significant prognostic value (7). More recently, single-cell RNA sequencing (scRNA-seq) has uncovered profound fibroblast heterogeneity, dynamic immune cell interactions, and activation of fibrosis-associated signaling networks within keloid tissue (8). Nevertheless, effective targeted therapies remain scarce, and robust biomarker systems for early prediction have yet to be established.

Autophagy is a lysosome-dependent intracellular degradation process that plays a critical role in maintaining cellular metabolic homeostasis, modulating stress responses, and regulating tissue remodeling. In highly fibrotic conditions such as keloids, autophagic activity is often dysregulated, leading to aberrant fibroblast proliferation and excessive extracellular matrix (ECM) accumulation (9). Notably, autophagy exerts a dual role—both protective and pathogenic—in keloid pathogenesis. On one hand, moderate activation of autophagy can suppress fibrosis; for example, autophagy induction has been shown to downregulate Notch1 and the NLRP3 inflammasome, thereby reducing chronic inflammation and myofibroblast activation (10). On the other hand, impaired autophagic flux or excessive autophagy may exacerbate lesion progression, as evidenced by IL-17 signaling–mediated autophagy inhibition, which promotes necrotic cell death and collagen deposition (11).

Given that the regulatory network of autophagy in keloids remains incompletely understood and that key molecular targets have not been fully elucidated, this study aimed to systematically identify autophagy-related genes (ARGs) potentially implicated in keloid formation. By integrating differential gene expression analysis, WGCNA, and multiple machine learning algorithms, we screened for ARGs closely associated with keloid pathology. Ultimately, this study sought to explore the potential regulatory roles of key autophagy-related genes in keloid-associated fibrosis and to provide a theoretical foundation and hypothesis-generating molecular candidates for future mechanistic studies and targeted therapeutic intervention.

## 2 Materials and methods

### 2.1 Data acquisition and differential expression analysis

Using the keyword “keloid”, we searched the Gene Expression Omnibus (GEO, https://www.ncbi.nlm.nih.gov/geo/) for human bulk transcriptomic datasets. Five datasets were retrieved: GSE44270 (9 keloid, 3 controls), GSE145725 (9 keloid, 10 controls), GSE7890 (5 keloid, 5 controls), GSE218007 (23 keloid, 6 controls), and GSE280420 (3 keloid, 3 controls). Among these, GSE44270 and GSE145725 were designated as the discovery cohort. For each discovery dataset, raw expression values were log_2_-transformed where appropriate according to quantile distributions and normalized using the normalizeBetweenArrays function in the limma R package. For genes mapped by multiple probes, averaged expression values were calculated using the avereps function (12). The two discovery datasets were then merged on shared gene symbols, and batch effects were corrected with the ComBat function in the sva package, which implements an empirical Bayes framework (13).

The remaining three datasets were initially considered as independent validation cohorts. GSE7890, which contains both keloid and normal skin samples with complete expression information for the target diagnostic genes, was used as the primary external validation set to evaluate the expression patterns and diagnostic performance of ADRB2 and PLK2. In GSE218007, several of the candidate diagnostic genes were not represented or lacked reliable probe annotation; therefore, this dataset could not be used for validating the full diagnostic gene set and was only employed in preliminary exploratory analyses rather than in the final validation of ADRB2 and PLK2. GSE280420 has a very small sample size (3 keloid vs. 3 controls) and, in our analyses, yielded relatively low and unstable diagnostic performance for the candidate genes; as a result, it was not included as a main validation cohort but was retained as a sensitivity reference. The role of each dataset in the overall analysis pipeline (discovery, filtering, and external validation) is summarized in the bioinformatics workflow diagram (flow chart). Autophagy-related genes (ARGs) were obtained from the Human Autophagy Database (HADb, https://www.autophagy.lu/) and the Molecular Signatures Database (MSigDB, https://www.gsea-msigdb.org/gsea/msigdb/).

(Workflow of transcriptomic data acquisition, preprocessing, and validation strategy.

**Figure 1:**
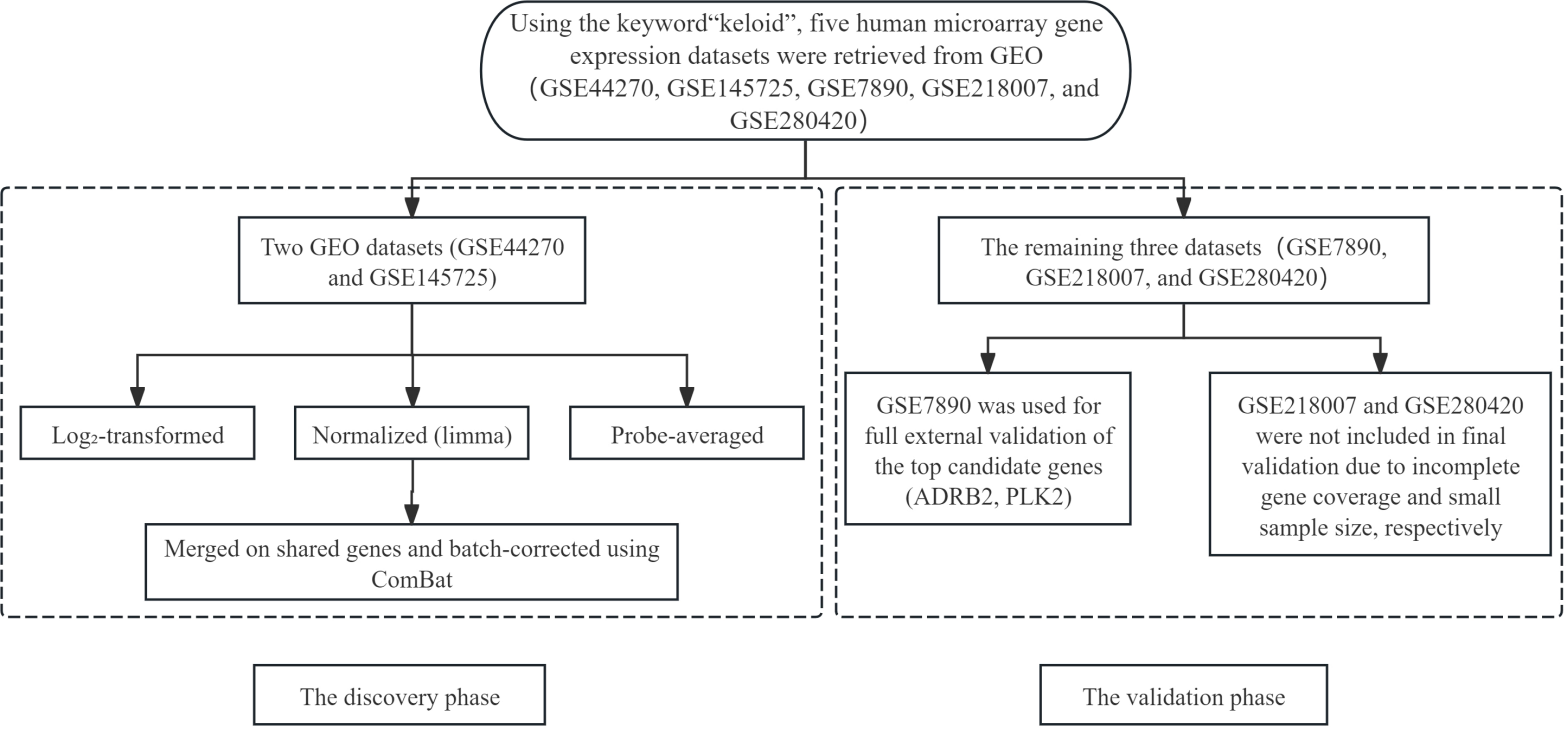
Workflow of transcriptomic data acquisition, preprocessing, and validation strategy. Human keloid-related microarray datasets were retrieved from the Gene Expression Omnibus (GEO) database using the keyword “keloid,” including GSE44270, GSE145725, GSE7890, GSE218007, and GSE280420. GSE44270 and GSE145725 were selected as discovery cohorts and underwent log transformation, normalization using the limma package, probe-level averaging, and batch-effect correction with ComBat before downstream analyses. The remaining datasets were designated for validation: GSE7890 was used for external validation of the top candidate genes (ADRB2 and PLK2), whereas GSE218007 and GSE280420 were excluded from final validation due to incomplete gene coverage and small sample size, respectively. This workflow delineates the discovery and validation phases of the integrated transcriptomic analysis.

Human keloid-related microarray datasets were retrieved from the Gene Expression Omnibus (GEO) database using the keyword “keloid, “ including GSE44270, GSE145725, GSE7890, GSE218007, and GSE280420. GSE44270 and GSE145725 were selected as discovery cohorts and underwent log transformation, normalization using the limma package, probe-level averaging, and batch-effect correction with ComBat before downstream analyses. The remaining datasets were designated for validation: GSE7890 was used for external validation of the top candidate genes (ADRB2 and PLK2), whereas GSE218007 and GSE280420 were excluded from final validation due to incomplete gene coverage and small sample size, respectively. This workflow delineates the discovery and validation phases of the integrated transcriptomic analysis) **Figure 1**.

### 2.2 Identification of differentially expressed genes

Differential expression analysis between keloid and control samples was performed using the limma package in R. A linear model was first fitted for each gene, followed by empirical Bayes moderation (eBayes) to estimate the statistical significance of expression differences. Differentially expressed genes (DEGs) were defined using the following thresholds: absolute log_2_ fold change (|log_2_FC|) > 0.5 and false discovery rate (FDR) < 0.05 (14). The resulting DEGs were visualized using heatmaps and volcano plots generated with the pheatmap and ggplot2 packages, respectively.

### 2.3 Weighted gene co-expression network analysis

Weighted gene co-expression network analysis was performed using the WGCNA R package (v1.72) to identify co-expression modules associated with keloids (15). The expression matrix was normalized, and low-variance genes (standard deviation ≤ 0.5) and samples identified as outliers by hierarchical clustering were removed. A scale-free topology analysis using the pickSoftThreshold function was conducted over a range of candidate soft-thresholding powers (β = 1–20). A soft-thresholding power of β = 17 was selected as the smallest value at which the scale-free topology fit index (R²) exceeded 0.85 while maintaining adequate mean connectivity, and it also yielded a clearer separation of branches in the resulting gene dendrogram, indicating a more stable and well-structured network topology. The resulting adjacency matrix was then transformed into a topological overlap matrix (TOM). Gene modules were identified using the dynamic tree-cutting algorithm (minModuleSize = 40, deepSplit = 2), and similar modules were merged using a module eigengene correlation threshold of 0.6. Module–trait relationships were calculated to identify keloid-associated modules. Within these modules, genes with high module membership (MM > 0.8) and gene significance (GS > 0.5) were defined as candidate intramodular hub genes for further analysis.

### 2.4 Gene ontology and kyoto encyclopedia of genes and genomes enrichment analysis

GO and KEGG enrichment analyses were performed for the 10 candidate genes using the clusterProfiler package (v4.2.2) in R (16). GO analysis covered three functional categories: biological process (BP), cellular component (CC), and molecular function (MF), with Homo sapiens specified as the background reference. The Benjamini–Hochberg method was applied to adjust for multiple testing, and GO terms with adjusted p-values (p.adjust) < 0.05 were considered statistically significant. Similarly, KEGG pathway analysis was performed using a significance threshold of p.adjust < 0.05. Significant enrichment results were visualized using bubble plots, chord diagrams, and bar plots.

### 2.5 Biomarker selection using machine learning algorithms

To identify robust candidate biomarkers, three machine learning algorithms were applied in parallel: random forest (RF), least absolute shrinkage and selection operator (LASSO), and support vector machine–recursive feature elimination (SVM-RFE). LASSO regression was performed using the glmnet package with 10-fold cross-validation to determine the optimal regularization parameter (lambda.min), and genes with non-zero coefficients at lambda.min were selected as potential features (17). SVM-RFE was implemented using the msvmRFE.R script, with 10-fold cross-validation used to rank genes based on their recursive feature elimination scores (18). For RF analysis, the randomForest package was used to construct a classification model, and genes were ranked according to their variable importance scores (19). The intersection of the gene sets identified by all three algorithms was defined as the consensus set of candidate biomarkers.

### 2.6 Diagnostic performance evaluation

Receiver operating characteristic (ROC) curve analysis was performed using the pROC and glmnet packages to evaluate the diagnostic performance of individual genes and multi-gene combinations. The area under the curve (AUC) and corresponding 95% confidence intervals (CIs) were calculated (20). A logistic regression model was constructed using the rms and rmda packages. A nomogram was generated to visualize the predictive model, and calibration curves were plotted using bootstrap resampling (B = 1000) to assess model calibration. Clinical utility was further evaluated through decision curve analysis (DCA) to estimate the net clinical benefit across a range of threshold probabilities. The Wilcoxon rank-sum test was applied to assess statistical differences between groups.

### 2.7 Immune infiltration analysis

Single-sample gene set enrichment analysis (ssGSEA) was performed using the GSVA package (v1.44.5) in R to estimate enrichment scores for immune cell–related and immune pathway–related gene signatures (21). Genes with missing values were excluded, and duplicate probes were collapsed using the avereps function. Immune-related gene sets were imported via the getGmt function, and enrichment scores were calculated using the gsva function with parameters set as kcdf = “Gaussian” and abs.ranking = TRUE. The resulting enrichment scores were linearly normalized to a 0–1 range for downstream visualization, between-group comparisons, and correlation analyses with candidate gene expression levels.

### 2.8 Patient sample collection

A total of four keloid tissue samples and four paired adjacent normal skin samples were obtained from patients diagnosed with keloids at the Department of Dermatology, Affiliated Hospital of Yanbian University, in 2025. All patients voluntarily underwent surgical excision and met the following inclusion criteria: age between 18 and 45 years; absence of local infection at the lesion site; no prior treatment with medication, radiation, or laser therapy; and no history of systemic organic disease. All tissue samples were collected after obtaining written informed consent from each participant. The study protocol was reviewed and approved by the Medical Ethics Committee of the Affiliated Hospital of Yanbian University (Approval No. 2024359). Each specimen was divided into two portions: one was fixed in formalin and paraffin-embedded for histological analysis, and the other was preserved for protein extraction.

### 2.9 Hematoxylin and eosin staining, Masson’s trichrome staining, and immunohistochemistry

Keloid and adjacent normal skin tissues were fixed in 4% paraformaldehyde, dehydrated, and paraffin-embedded. Sections were cut at a thickness of 3 μm. For H&E staining, paraffin sections were deparaffinized in xylene (15 min × 2), rehydrated through a graded ethanol series (100%, 95%, 85%, 75%, 3 min each), stained with hematoxylin for 20 min, differentiated in 1% acid alcohol for 15 s, and counterstained with eosin for 30 s. Slides were then dehydrated, cleared, and mounted. For Masson’s trichrome staining, rehydrated sections were treated with Bouin’s solution at 56°C for 1 h, followed by nuclear staining with Weigert’s iron hematoxylin, cytoplasmic staining with Biebrich scarlet–acid fuchsin, differentiation with phosphotungstic/phosphomolybdic acid, and collagen fiber staining with aniline blue (methyl blue). Slides were then dehydrated and mounted. For immunohistochemistry (IHC), sections were deparaffinized, rehydrated, and washed with PBS. Antigen retrieval was performed under high-temperature conditions using citrate buffer, followed by blocking of endogenous peroxidase with 3% H_2_O_2_. Non-specific binding was blocked with 10% donkey serum at room temperature for 1 h. Primary antibodies (PLK2: 1:100, BY3028, Abways; ADRB2: 1:100, CY6689, Abways) were applied and incubated overnight at 4°C. The next day, sections were incubated with signal enhancement reagent and horseradish peroxidase (HRP)-conjugated secondary antibody, followed by DAB chromogenic detection and hematoxylin counterstaining. After dehydration and clearing, sections were mounted. Images were acquired and analyzed using ImageJ software, with three random fields selected per section for quantitative assessment.

### 2.10 Western blot analysis

Total protein was extracted from four keloid tissues and four paired adjacent normal skin tissues using RIPA lysis buffer supplemented with protease and phosphatase inhibitors. Protein concentrations were determined, and equal amounts of protein were separated by SDS-PAGE and transferred onto PVDF membranes. After blocking at room temperature for 1 h, membranes were incubated overnight at 4°C with primary antibodies against PLK2 (1:1000, BY3028, Abways), ADRB2 (1:1000, CY6689, Abways), and GAPDH (1:5000, AP0066, Bioworld). Membranes were then washed with PBST (3 × 10 min) and incubated with HRP-conjugated secondary antibodies (Alexa Fluor 790–conjugated AffiniPure Donkey Anti-Rabbit IgG (H+L), 1:10000, 711-655-152, Jackson ImmunoResearch Laboratories) at room temperature for 2 h. After additional washing with PBST (2 × 10 min) and PBS (10 min), signals were developed. Bands were visualized using an Odyssey CLx imaging system, and band intensities were quantified using ImageJ software.

### 2.11 Cell culture and autophagy modulation

Normal human dermal fibroblasts (NFs; CRL-4066) and keloid-derived fibroblasts (KFs; CRL-1762) were obtained from ATCC (USA). Cells were cultured in high-glucose DMEM (L100, BaiDi Biotechnology) supplemented with 10% fetal bovine serum (F801, BaiDi Biotechnology) and 1% penicillin–streptomycin (A200, BaiDi Biotechnology) at 37°C in 5% CO_2_. Cells at passages 3–6 were used for experiments.

To modulate autophagy, KFs were treated with Earle’s Balanced Salt Solution (EBSS; C0213, Beyotime) to induce autophagy or chloroquine (CQ; MedChemExpress) to inhibit autophagic flux. Untreated KFs served as controls. Cells were subsequently collected for Western blotting, immunofluorescence, and functional analyses.

### 2.12 Autophagy fluorescence detection

Autophagic activity was assessed using an monodansylcadaverine (MDC)-based autophagy detection kit (C3019S, Beyotime) according to the manufacturer’s instructions. Cells were incubated with MDC working solution at 37°C for 30 min in the dark, washed, and imaged immediately under a fluorescence microscope. MDC-positive autophagic vacuoles were quantified using ImageJ.

### 2.13 Western blot analysis

Total protein was extracted using RIPA buffer containing protease and phosphatase inhibitors. Equal amounts of protein were separated by SDS–PAGE, transferred to PVDF membranes, and blocked for 1 h at room temperature. Membranes were incubated overnight at 4°C with primary antibodies against LC3 (1:1000, CY5992, Abways), p62 (1:1000, CY5546, Abways), ADRB2 (1:1000, AY1109, Abways), PLK2 (1:1000, BY3028, Abways), and GAPDH. After incubation with HRP-conjugated secondary antibodies, signals were visualized by chemiluminescence and quantified using ImageJ, with normalization to GAPDH.

### 2.14 Statistical analysis

All statistical analyses were performed using R software (version 4.3.2) and GraphPad Prism 8. For experimental data derived from H&E staining, Masson trichrome staining (% area), and Western blot (WB) densitometry, continuous variables were first inspected visually using histograms and Q–Q plots, and formal normality was assessed with the Shapiro–Wilk test. When the data approximately conformed to a normal distribution and showed comparable variances between groups, results were expressed as mean ± standard error of the mean (SEM), and differences between keloid and normal skin were evaluated using two-tailed unpaired Student’s t-tests (parametric test). When the normality assumption was clearly violated and could not be improved by simple transformation, the non-parametric Mann–Whitney U test was applied instead. Thus, parametric tests were preferred when their assumptions were met to maximize statistical power, whereas non-parametric tests were reserved for skewed or non-normally distributed data.

For correlation analyses in transcriptomic data, Pearson correlation coefficients were calculated when both variables were approximately normally distributed; otherwise, Spearman rank correlation coefficients were used. For high-dimensional gene expression analyses, p-values were adjusted for multiple testing using the Benjamini–Hochberg false discovery rate (FDR) procedure, with FDR < 0.05 considered statistically significant. Unless otherwise specified, all statistical tests were two-tailed, and p < 0.05 was regarded as statistically significant. Levels of significance are denoted as follows: p < 0.05 (*), p < 0.01 (**), and p < 0.001 (***).

## 3 Results

### 3.1 Differential expression analysis of keloid-associated genes

**Figure 2:**
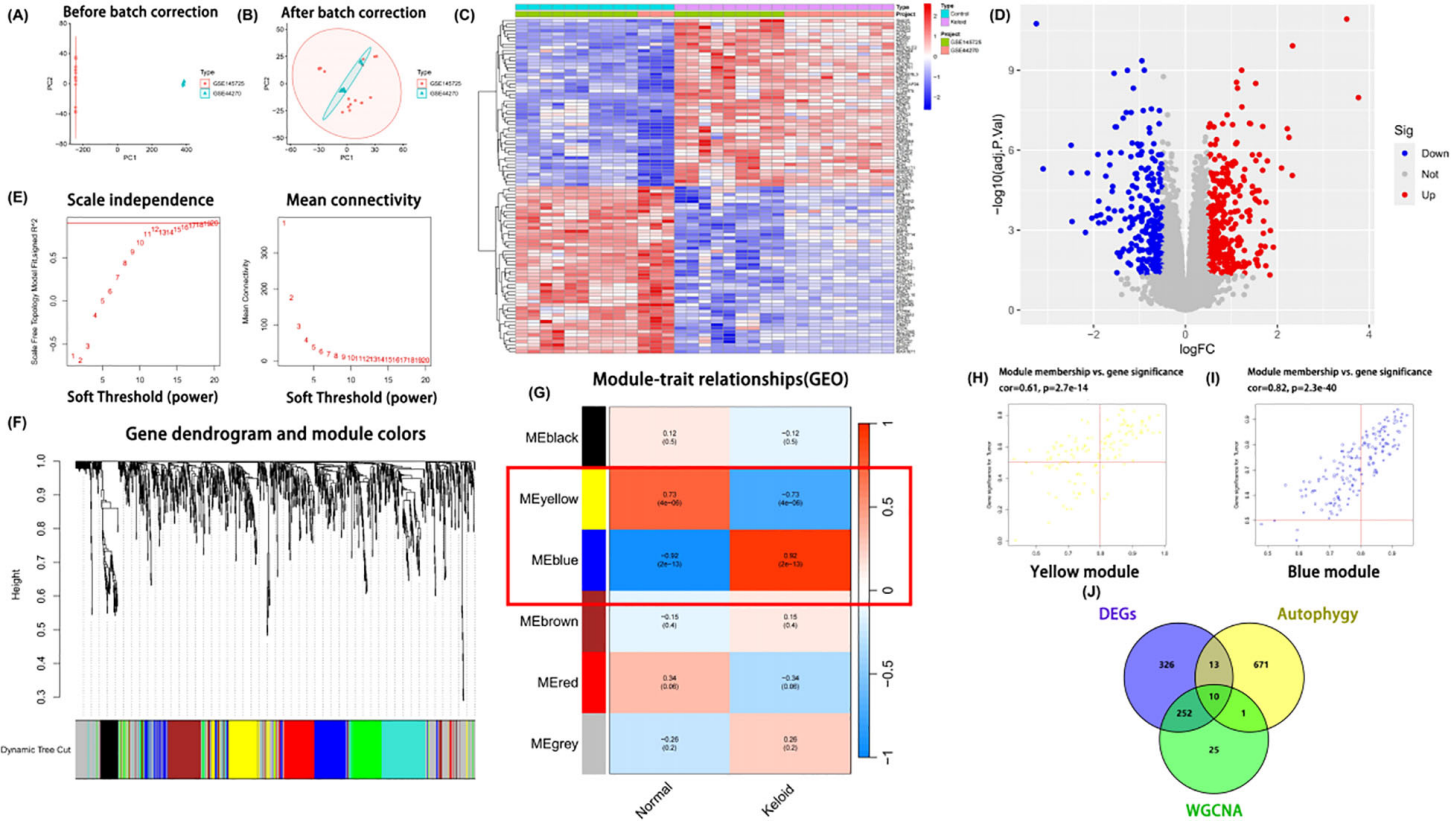
Results of differential expression analysis and weighted gene co-expression network analysis (WGCNA). **(A, B)** Principal component analysis (PCA) plots showing sample distributions before and after batch-effect correction. **(C)** Heatmap displaying the expression patterns of differentially expressed genes (DEGs) across all samples. **(D)** Volcano plot illustrating significantly upregulated (red) and downregulated (blue) DEGs. **(E)** Scale-free topology model fit and mean connectivity for different soft-thresholding powers used in network construction. **(F)** Gene dendrogram and corresponding module detection based on hierarchical clustering. **(G)** Module–trait correlation heatmap; red indicates a positive correlation and blue indicates a negative correlation. **(H, I)** Scatter plots of gene significance (GS) versus module membership (MM) for genes in modules significantly associated with keloids. **(J)** Venn diagram showing the overlap among DEGs, autophagy-related genes, and WGCNA module genes.

To investigate the differential expression of autophagy-related genes in keloids, the GSE44270and GSE145725 datasets were first subjected to rigorous quality assessment. For each dataset, raw expression values were visualized using boxplots to inspect signal distributions and detect potential outliers (GSE44270: **Supplementary Figure S1A**; GSE145725: **Supplementary Figure S1C**). After log_2_ transformation, within-dataset normalization was performed using thenormalizeBetweenArrays function in the limma package, and post-normalization boxplots indicated markedly improved distribution uniformity (GSE44270: **Supplementary Figure S1B**; GSE145725: **Supplementary Figure S1D**). Subsequently, the two datasets were merged based on shared gene symbols. Quality controlof the combined dataset revealed obvious platform-related differences in expression distributions, as shown by the pre-correction boxplot (**Supplementary Figure S1E**) and principal component analysis (PCA), in which samples clustered predominantly by dataset (**Figure 2A**). Batch effects were then adjusted using the ComBat algorithm implemented in the svapackage, with dataset origin specified as the batch variable. After correction, the boxplot of the merged dataset demonstrated a stable and homogeneous distribution across all arrays (**Supplementary Figure S1F**), and PCA showed improved mixing of keloid and control samples from both datasets (**Figure 2B**), indicating that non-biological batch variation had been effectively minimized.

On this merged and batch-corrected expression matrix, differential expression analysis identified a total of 601 differentially expressed genes (DEGs) under the thresholds |log_2_FC| > 0.5 and FDR < 0.05, including 335 upregulated and 266 downregulated genes (**Supplementary Table S1**). Hierarchical clustering of the top DEGs revealed clear separation between keloid and normal tissues (**Figure 2C**), while the volcano plot depicted the overall distribution of up- and down-regulated genes (**Figure 2D**). These DEGs provided a robust pool of candidate genes for subsequent exploration of autophagy involvement in keloid pathogenesis.

### 3.2 WGCNA identifies keloid-associated differentially expressed genes

Based on the 601 differentially expressed genes (DEGs), a weighted gene co-expression network wasconstructed using the WGCNA package in R. Prior to network construction, sample clustering confirmedthat no obvious outliers needed to be removed (**Supplementary Figure S2**). A series of candidate soft-thresholding powers (β = 1–20) were then evaluated using the pickSoftThreshold function. For each power, the scale-free topology fit index (signed R²) and mean connectivity were calculated (**Figure 2E**). We required the network to approximate a scale-free topology with a signed R² ≥ 0.85 while maintaining sufficient mean connectivity to avoid an overly sparse network. Among the tested powers, β = 17 was the smallest value at which the signed R² first exceeded this threshold and the mean connectivity curve began to plateau, and it also produced clearer branch separation and more distinct module boundaries in the subsequent gene dendrogram, indicating a more stable network structure; therefore, β = 17 was selected as the soft-thresholding power for subsequent analyses (**Figure 2E**). Using this soft threshold, an adjacency matrix was constructed and transformed into a topological overlap matrix (TOM). Hierarchical clustering based on the TOM, followed by dynamic tree cutting and module merging, identified six distinct gene modules (**Figure 2F**). The resulting module structure was biologically interpretable and provided the basis for downstream analyses of module–trait relationships in keloid pathogenesis.

Module–trait correlation analysis revealed that the yellow module (r = 0.73, p = 4 × 10^-6^) and blue module (r = 0.92, p = 2 × 10^-13^) were strongly positively correlated with the keloid phenotype (**Figure 2G**), encompassing a total of 288 candidate genes (**Supplementary Table S2**). The relationship between module membership (MM) and gene significance (GS) further supported the importance of the yellow (r = 0.61, p = 2.7 × 10^-14^; **Figure 2H**) and blue (r = 0.82, p = 2.3 × 10^-40^; **Figure 2I**) modules in keloid pathology.

Intersecting the genes from these two modules with autophagy-related gene sets (sourced from HADb and MSigDB) and the DEGs resulted in the identification of 10 candidate hub genes: ADRB2, PLK2, EPAS1, KCNB1, PLIN2, SLC7A5, LEPR, SNCAIP, SVIP, and QSOX1 (**Figure 2J**). These genes may be involved in autophagy dysregulation and immune modulation in keloids and were therefore selected as candidate targets for subsequent exploratory analyses and functional validation.

### 3.3 GO and KEGG enrichment analysis

**Figure 3:**
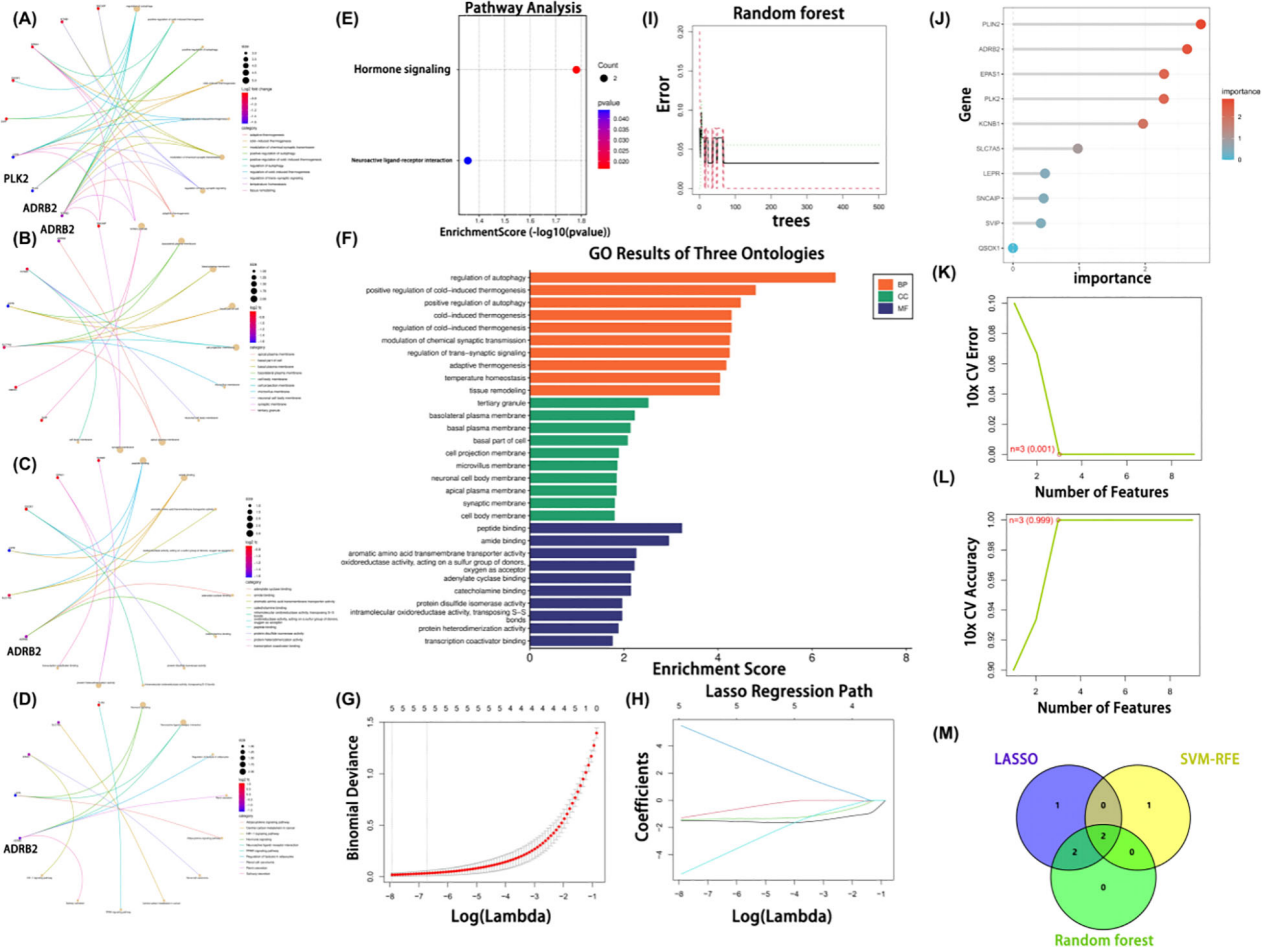
Functional enrichment analysis and machine learning–based selection of candidate genes. **(A–D)** Chord plots showing Gene Ontology (GO) enrichment results for the candidate genes, including biological process (BP), cellular component (CC), molecular function (MF), and KEGG pathway analysis. **(E)** KEGG enrichment bubble plot highlighting significantly enriched signaling pathways. **(F)** Bar plot summarizing GO enrichment results across the BP, CC, and MF categories. **(G, H)** LASSO regression analysis: feature selection process and cross-validation curve for the optimal lambda value. **(I)** Error rate curve of the random forest model across different numbers of trees. **(J)** Ranking of gene importance scores from the random forest model. **(K, L)** SVM-RFE analysis showing the relationship between the number of features and cross-validation error. **(M)** Venn diagram illustrating the intersection of genes selected by the LASSO, SVM-RFE, and random forest algorithms, identifying ADRB2 and PLK2 as core hub gene candidates.).

To explore the functional characteristics of the 10 candidate genes, Gene Ontology (GO) and Kyoto Encyclopedia of Genes and Genomes (KEGG) enrichment analyses were performed. In the GO biological process (BP) category, the genes were mainly enriched in processes related to the regulation of autophagy, cold-induced thermogenesis, and modulation of chemical synaptic transmission, with regulation of autophagy showing the highest enrichment significance (enrichment score > 6, p < 2.5 × 10^-5^; **Figure 3A**; **Supplementary Figure S3A**). Cellular component (CC) analysis indicated that these genes were primarily localized to tertiary granules, the basolateral plasma membrane, and the cell base, with tertiary granule showing the most significant enrichment (enrichment score > 2.4, p < 0.006; **Figure 3B**; **Supplementary Figure S3B**). For molecular function (MF), significant enrichment was observed in terms such as peptide binding, amide binding, and transmembrane transport of aromatic amino acids, among which peptide binding (enrichment score > 3.0, p < 0.004) and oxidoreductase activity were the most prominent (**Figure 3C**; **Supplementary Figure S3C**).

KEGG pathway analysis revealed that the candidate genes were associated with hormone signaling pathways, neuroactive ligand–receptor interaction, and regulation of lipolysis in adipocytes. Among these, the first two pathways were statistically significant (p = 0.020 and 0.040, respectively; **Figures 3D, E**). A combined bar plot summarizing all three GO categories further highlighted the potential roles of these genes in autophagy, thermogenic responses, subcellular structural localization, and molecular transport (**Figure 3F**). Collectively, these findings suggest that the candidate genes may be functionally involved in autophagy regulation, signal transduction, and intracellular material transport, and may contribute to the pathogenesis of keloids through hormonal and neuroactive signaling mechanisms.

### 3.4 Identification of autophagy-related hub genes via machine learning

To systematically identify key autophagy-related regulators potentially associated with the development and progression of keloids, three supervised machine learning algorithms—Least Absolute Shrinkage and Selection Operator (LASSO) logistic regression, Support Vector Machine–Recursive Feature Elimination (SVM-RFE), and Random Forest (RF)—were applied to the ten previously selected candidate genes. All models were implemented in R using the glmnet, e1071, and randomForest packages.

For LASSO regression, all predictors were standardized to zero mean and unit variance. A sequence of 100 logarithmically spaced regularization parameters (λ) was generated using the glmnet default settings, and model performance was evaluated by 10-fold cross-validated binomial deviance. The optimal λ (λ_min) was defined as the value that minimized the mean cross-validated deviance (**Figure 3G**). The corresponding coefficient profiles illustrated the progressive shrinkage of gene coefficients with increasing λ (**Figure 3H**), and five genes with non-zero coefficients at λ_min—ADRB2, EPAS1, KCNB1, PLIN2, and PLK2—were retained. For the RF model, an ensemble of 500 decision trees (ntree = 500) was grown, with the number of variables randomly sampled at each split set to mtry = 3. All other parameters were kept at their default values. Variable importance was quantified using the mean decrease in Gini index, and four genes with importance scores > 2—PLIN2, ADRB2, EPAS1, and PLK2—were selected (**Figures 3I, J**). For SVM-RFE, a linear-kernel support vector machine was used as the base classifier. At each iteration, the feature with the smallest absolute weight was removed, and classification performance for a given feature subset was assessed by 10-fold cross-validation. The feature set with the highest cross-validated accuracy (accuracy = 0.999, error = 0.001) was chosen, yielding three top-ranked genes: PLK2, LEPR, and ADRB2 (**Figures 3K, L**).

Finally, a Venn diagram integrating the results of all three algorithms (**Figure 3M**) showed that ADRB2 and PLK2 were consistently selected across LASSO, RF, and SVM-RFE. On this basis, ADRB2 and PLK2 were prioritized as core autophagy-related hub gene (ARG) candidates and were selected for subsequent exploratory functional validation and mechanistic studies.

### 3.5 Diagnostic performance evaluation of ARGs in keloids

**Figure 4:**
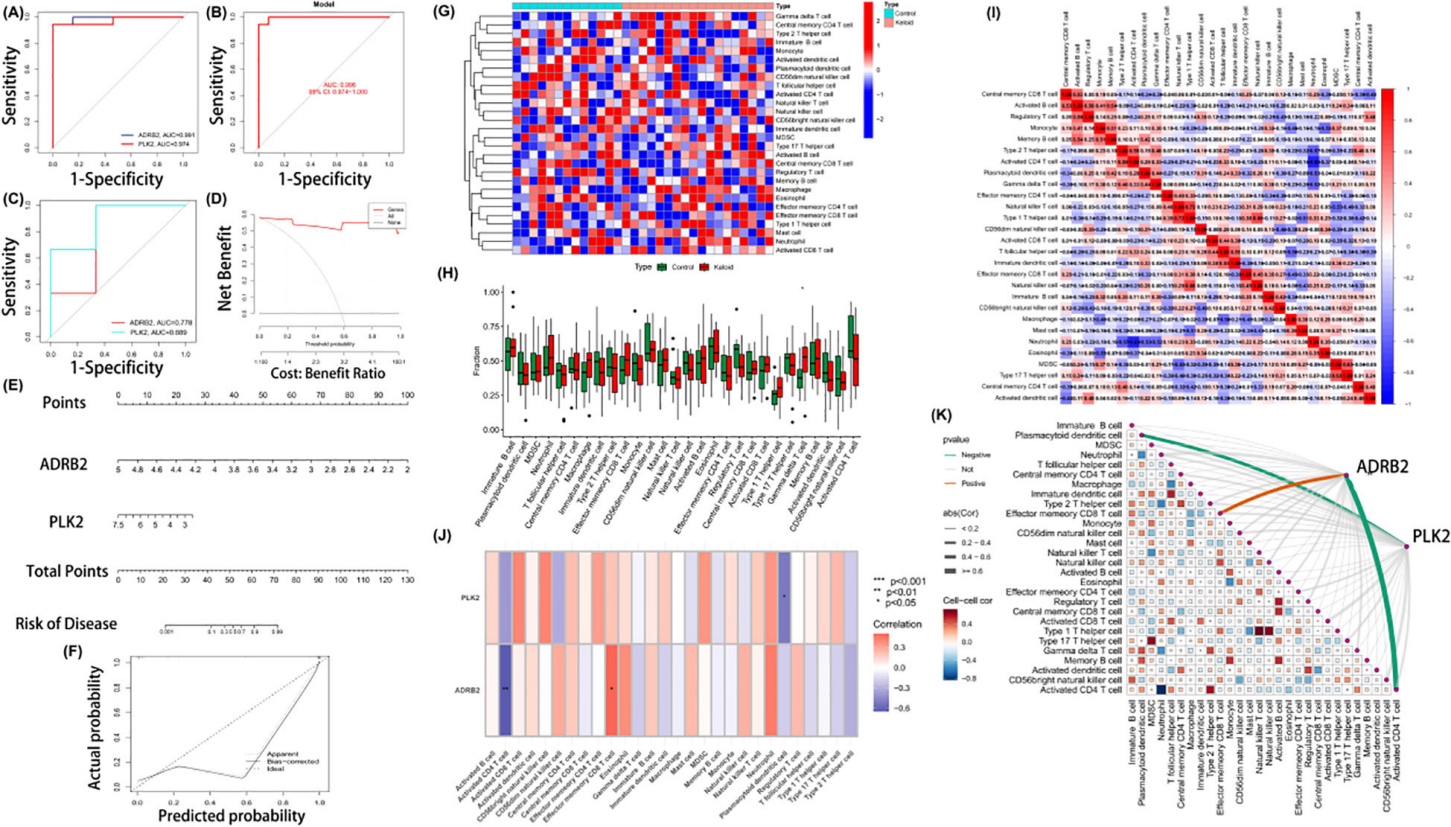
Evaluation of diagnostic performance and immune-related transcriptional patterns associated with hub genes. **(A–C)** ROC curves and corresponding AUC values for ADRB2, PLK2, and their combination in the discovery (training) and external validation cohorts. **(D)** Decision curve analysis (DCA) showing the net clinical benefit of the two-gene model across a range of threshold probabilities. **(E)** Nomogram based on ADRB2 and PLK2 expression scores for predicting keloid risk. **(F)** Calibration curve assessing the agreement between predicted probabilities and observed outcomes. **(G)** Correlation heatmap of ssGSEA-derived immune-related gene signature scores. **(H)** Boxplots comparing ssGSEA enrichment scores for immune cell–related signatures between keloid and normal samples. **(I)** Heatmap of immune-related ssGSEA enrichment scores across all samples, grouped by tissue type. **(J)** Heatmap showing correlations between ADRB2, PLK2, and immune cell–related ssGSEA signatures. **(K)** Network plot illustrating the relationships between ADRB2, PLK2, and immune cell–related or immune pathway–related ssGSEA signatures.

To further explore the diagnostic potential of the autophagy-related candidate genes ADRB2 and PLK2 in keloids, we performed receiver operating characteristic (ROC) curve analysis in the discovery (training) set. In this cohort, ADRB2 and PLK2 showed high apparent diagnostic discrimination, with areas under the curve (AUCs) of 0.991 and 0.974, respectively (**Figure 4A**). A combined two-gene model incorporating both markers achieved an AUC of 0.996 (95% CI: 0.974–1.000) (**Figure 4B**). Given the limited sample size, these values are likely optimistic estimates of performance and may reflect a degree of overfitting.

To partially assess generalizability, we applied the same two-gene model to an independent external dataset (GSE7890). In this validation cohort, the diagnostic performance was more modest, with AUCs of 0.778 for ADRB2 and 0.889 for PLK2 (**Figure 4C**), suggesting that the model retains discriminatory ability but that the performance observed in the training set may overestimate its true accuracy. Decision curve analysis (DCA) indicated that, within the studied datasets, the two-gene model could provide net clinical benefit across a range of threshold probabilities (**Figure 4D**), although these results should be interpreted cautiously in light of the small sample size. A nomogram based on ADRB2 and PLK2 expression scores showed a monotonic increase in predicted keloid risk with higher total scores (predicted probability range: 0.001–0.99) (**Figure 4E**), and calibration curves demonstrated reasonable agreement between predicted probabilities and observed outcomes in the discovery cohort (**Figure 4F**).

Overall, these analyses suggest that ADRB2 and PLK2 have potential value as candidate diagnostic biomarkers and that a simple two-gene model may offer discriminatory capacity for keloids. However, the current model should be regarded as exploratory and hypothesis-generating, and its diagnostic performance and clinical utility require validation in larger, independent cohorts to rigorously address the risk of overfitting.

### 3.6 Immune cell infiltration analysis

To explore immune-related transcriptional patterns in keloid tissue, we calculated single-sample Gene Set Enrichment Analysis (ssGSEA) scores for immune cell–associated gene signatures based on the GSE145725 and GSE44270 datasets (**Figure 4G**). Compared with normal skin, keloid samples showed lower ssGSEA enrichment scores for signatures related to immature dendritic cells (iDCs), natural killer (NK) cells, and regulatory T cells (Tregs), and higher scores for signatures related to neutrophils, macrophages, and effector memory CD8^+^ T cells (**Figure 4H**), indicating an altered immune-related gene expression profile rather than directly measured immune cell abundance. Correlation analyses of ssGSEA scores revealed a negative association between neutrophil-related and activated CD4^+^ T cell–related signatures and a positive association between Th1 cell–related signatures and those related to NK and NKT cells (**Figure 4I**), reflecting complex immune-related transcriptional relationships in keloid pathology.

Further analyses showed that ADRB2 expression was positively correlated with ssGSEA scores for effector memory CD8^+^ T cell–related signatures and negatively correlated with scores for activated CD4^+^ T cell– and Treg-related signatures, whereas PLK2 expression was negatively correlated with plasmacytoid dendritic cell (pDC)–related signatures (**Figure 4J**). A broader network analysis illustrated these correlations between both hub genes and multiple immune-related signatures (**Figure 4K**). Collectively, these ssGSEA-based findings suggest that ADRB2 and PLK2 expression is associated with immune-related transcriptional changes in keloid tissue. However, these results do not directly quantify immune cell abundance, activation status, or spatial distribution and should be considered hypothesis-generating; the proposed immune involvement of ADRB2 and PLK2 requires confirmation in dedicated immunological and functional experiments.

### 3.7 Validation of key biomarkers at the tissue level

**Figure 5:**
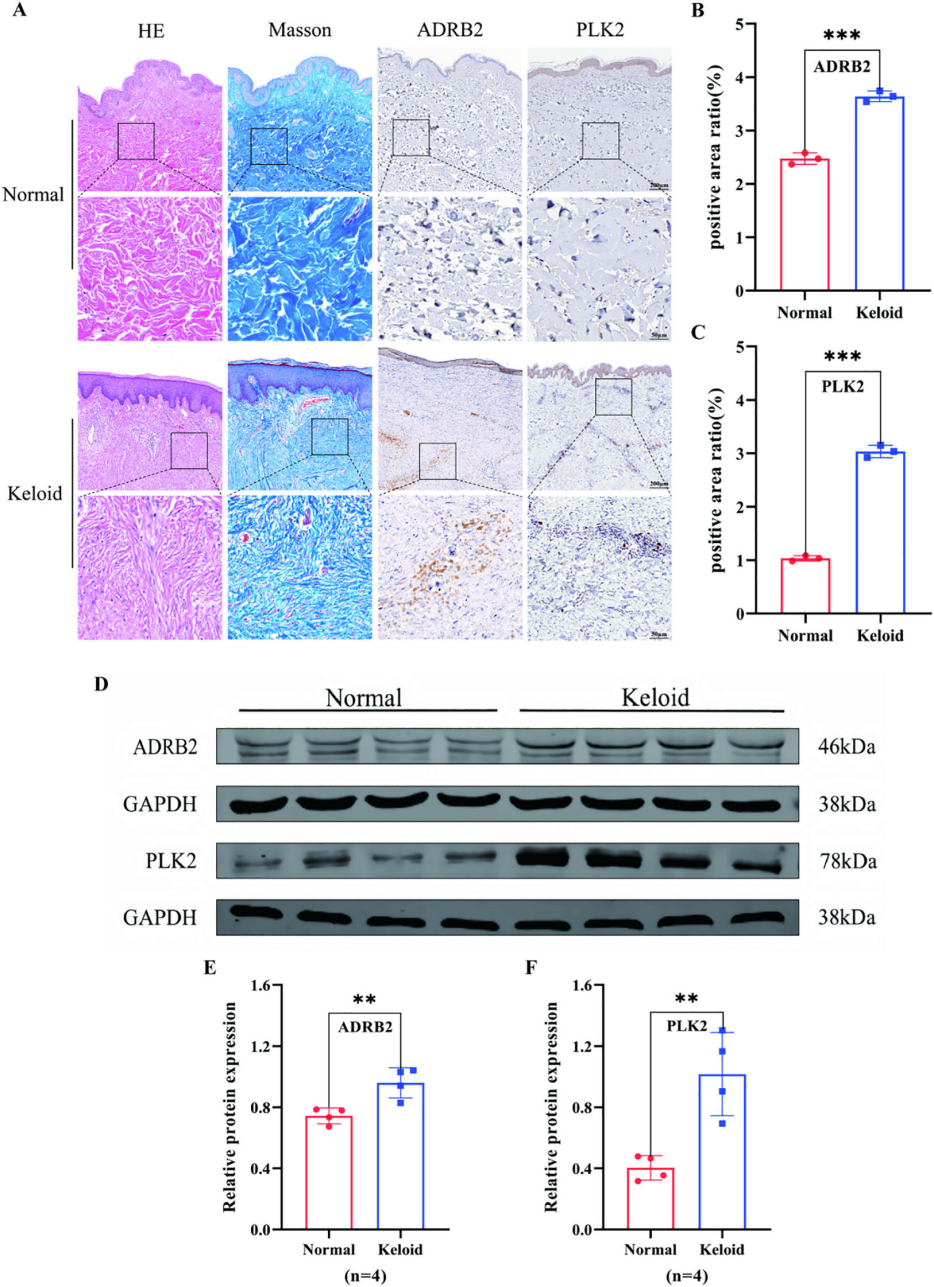
Upregulated expression of ADRB2 and PLK2 in keloid tissues. **(A)** H&E and Masson’s trichrome staining reveal dermal thickening and disorganized, densely packed collagen fibers in keloid tissues. Immunohistochemical staining shows increased positive expression of ADRB2 and PLK2 in keloid tissues, with ADRB2 predominantly localized to the epidermis and superficial dermis and PLK2 distributed throughout the dermal layer. **(B, C)** Quantitative analysis of IHC staining indicates that the positive staining areas of ADRB2 and PLK2 are significantly higher in keloid tissues than in normal controls (**p < 0.001). **(D)** Western blot analysis shows higher protein levels of ADRB2 and PLK2 in keloid tissues relative to normal skin, with GAPDH used as a loading control. **(E, F)** Densitometric analysis of WB bands demonstrates that ADRB2 and PLK2 protein expression levels are significantly increased in keloid tissues compared with normal controls (*p < 0.01; n = 4). p < 0.05 (*), p < 0.01 (**), and p < 0.001 (***).

Hematoxylin and eosin (H&E) and Masson’s trichrome staining revealed pronounced dermal thickening and disorganized, densely packed collagen fibers in keloid tissues, indicating marked histopathological features of hyperplasia and fibrosis suggestive of active tissue remodeling (**Figure 5A**, first and second columns). To assess the spatial expression patterns of the identified biomarkers, we performed immunohistochemical (IHC) staining for ADRB2 and PLK2. In normal skin, ADRB2 expression was weak and predominantly localized to the basal layer of the epidermis. In contrast, ADRB2 staining was more intense and extended into both the epidermis and superficial dermis in keloid tissues. Similarly, PLK2 showed only weak positivity in a limited number of dermal cells in normal skin, whereas in keloid tissues, PLK2 staining appeared stronger and more broadly distributed throughout the dermis (**Figure 5A**, third and fourth columns).

Quantitative image analysis of three random high-power fields per sample indicated that both ADRB2 and PLK2 had significantly higher positive staining areas in keloid tissues compared with normal controls in this small cohort (p < 0.001, **Figures 5B, C**). Western blotting was further conducted to evaluate the protein levels of ADRB2 and PLK2. Total protein was extracted from paired keloid and adjacent normal tissues from four patients, with GAPDH used as an internal control. In these samples, both ADRB2 and PLK2 showed higher protein expression in keloid tissues (**Figure 5D**), consistent with the IHC observations. Specifically, ADRB2 expression was increased by approximately 1.2-fold, and PLK2 expression by about 2.4-fold, both with statistically significant differences (p < 0.01, **Figures 5E–F**).

Taken together, these tissue-level observations are in line with the prior transcriptomic analyses—including differential expression analysis, WGCNA, and the three machine learning algorithms (LASSO, SVM-RFE, and random forest)—and support ADRB2 and PLK2 as autophagy- and immunity-related candidate diagnostic biomarkers and potential therapeutic research targets in keloids. However, given the limited sample size, these findings should be regarded as preliminary and require confirmation in larger, independent cohorts and dedicated functional studies.

### 3.8 Functional validation of ADRB2 and PLK2 in keloid fibroblasts via autophagy modulation

**Figure 6:**
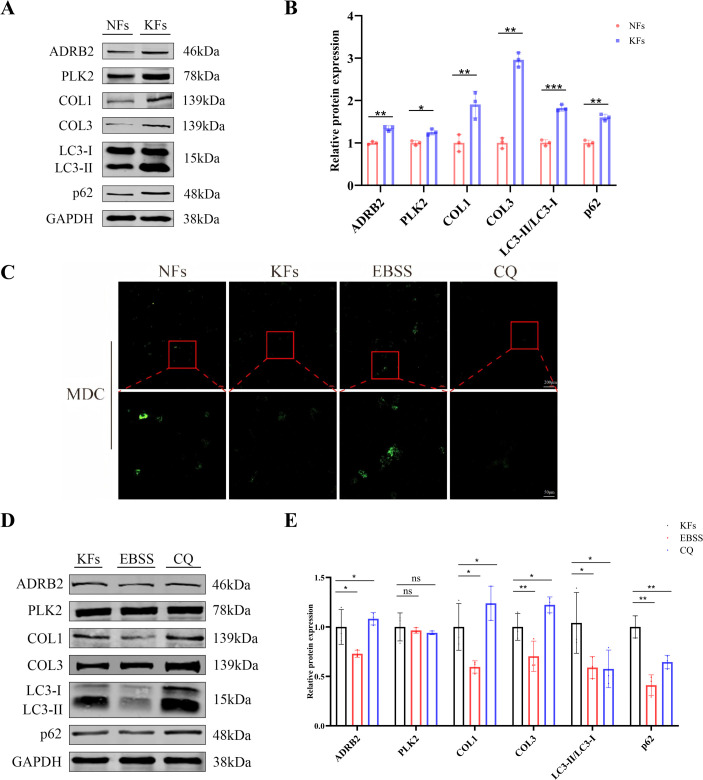
Functional validation of ADRB2 and PLK2 in keloid fibroblasts via autophagy modulation. **(A)** Western blot analysis comparing NFs and KFs shows increased protein expression of ADRB2, PLK2, COL1, COL3, LC3-II/LC3-I ratio, and p62 in KFs, with GAPDH used as a loading control. **(B)** Densitometric quantification of Western blot bands demonstrates significantly higher levels of ADRB2, PLK2, COL1, COL3, LC3-II/LC3-I, and p62 in KFs compared with NFs (*p < 0.05, **p < 0.01, ***p < 0.001). **(C)** Representative fluorescence images of MDC staining indicate reduced MDC-positive puncta in KFs compared with NFs, while EBSS treatment increases and CQ treatment decreases MDC-labeled autophagic structures. **(D)** Western blot analysis of KFs treated with EBSS or CQ shows that autophagy modulation alters fibrotic markers (COL1 and COL3), autophagy-related proteins (LC3-I/LC3-II and p62), and ADRB2 expression, whereas PLK2 levels remain relatively stable across treatments. **(E)** Quantitative analysis confirms that EBSS-induced autophagy activation reduces COL1, COL3, p62, and ADRB2 expression, while CQ-mediated autophagy inhibition results in increased levels of these proteins, with no significant change observed in PLK2 expression (ns, not significant; *p < 0.05, **p < 0.01).

To validate the cellular relevance of ADRB2 and PLK2, comparative experiments were conducted using NFs and KFs. At baseline, Western blot analyses revealed that KFs exhibited a pronounced fibrotic phenotype, characterized by significantly elevated protein expression of COL1 and COL3 compared with NFs (**Figures 6A, B**).

Baseline autophagy-related markers were also altered in KFs. Compared with NFs, KFs showed increased LC3-II/LC3-I ratios accompanied by marked accumulation of p62, together with reduced MDC-positive puncta, indicating altered autophagy-associated protein accumulation and impaired autophagic flux in keloid fibroblasts (**Figures 6A–C**). In parallel, ADRB2 and PLK2 protein levels were both significantly elevated in KFs relative to NFs, as confirmed by Western blot analyses (**Figures 6A, B**).

Autophagy modulation experiments were subsequently conducted in KFs. EBSS treatment effectively enhanced autophagy-associated fluorescence signals and reduced p62 levels, whereas CQ treatment led to further accumulation of autophagy-related signals and p62 (**Figures 6C–E**). Under these conditions, fibrotic marker expression was responsive to autophagy modulation. EBSS treatment significantly reduced COL1 and COL3 protein levels, while CQ treatment led to increased expression of these fibrotic markers compared with untreated KFs (**Figures 6D, E**). Notably, ADRB2 expression was sensitive to autophagy modulation, showing decreased levels following EBSS treatment and increased levels upon CQ treatment. In contrast, PLK2 protein expression remained relatively stable under both autophagy activation and inhibition conditions (**Figures 6D, E**).

These results demonstrate that keloid fibroblasts exhibit concurrent upregulation of fibrotic markers, autophagy-related proteins, and ADRB2/PLK2 at baseline, and that autophagy modulation differentially affects fibrotic markers and ADRB2 expression, while PLK2 expression remains stable under these conditions.

## 4 Discussion

Keloids are pathological scars that arise from aberrant wound healing, characterized by excessive fibroblast proliferation, ECM accumulation, and collagen overproduction, often resulting in considerable physical and psychological burden for affected individuals (22). With advances in cellular homeostasis research, autophagy—an essential mechanism in eukaryotic cells for maintaining intracellular equilibrium and eliminating damaged organelles and aberrant proteins—has been increasingly implicated in various fibrotic diseases (10). Dysregulated autophagy has been associated with uncontrolled fibroblast proliferation, excessive collagen deposition, and the persistent expansion of scar tissue. Acting as a double-edged sword, autophagy modulates inflammation, clears dysfunctional cellular components, and can either mitigate or exacerbate fibrosis depending on its activation status (23). These observations underscore the need to further clarify the interplay between autophagy and keloid pathogenesis through both bioinformatic and experimental approaches.

In this study, we leveraged transcriptomic data to perform an integrative analysis—combining multi-dataset profiling, WGCNA, and multiple machine learning algorithms—to prioritize autophagy-related hub gene candidates that may have regulatory roles in keloids. By intersecting results from differential gene expression analysis and WGCNA-derived co-expression modules, we identified ten candidate genes closely associated with autophagy. Subsequent GO and KEGG enrichment analyses revealed that these genes were primarily involved in biological processes such as “regulation of autophagy, “ “neuroactive ligand–receptor interaction, “ and “hormone signaling pathways, “ suggesting possible involvement in complex signaling and cellular homeostasis networks. Notably, the “regulation of autophagy” pathway exhibited the most significant enrichment, supporting the internal consistency of our analytic framework and indicating potential biological relevance of the selected targets.

To enhance the precision of feature selection and explore translational relevance, we employed a combinatorial approach integrating three widely used machine learning algorithms—LASSO, SVM-RFE, and random forest. This strategy yielded two consistently prioritized genes, ADRB2 and PLK2, which showed good diagnostic performance across both the training and external validation cohorts, suggesting that they may serve as candidate biomarkers. Functionally, ADRB2 and PLK2 have been implicated in regulating cellular metabolism, autophagy, and cell-cycle progression in several cell types, including fibroblasts and chondrocytes. Aberrant expression or signaling of these genes has been associated with altered autophagic flux, proliferation, and apoptosis, which in turn can influence tissue remodeling in fibrotic and degenerative conditions (24–27).

Further integrating ssGSEA-based immune-related transcriptional profiling, we observed a pronounced imbalance in immune-related gene signature patterns in keloid tissue. Pro-inflammatory signatures corresponding to neutrophils, M1 macrophages, and effector CD8^+^ T cells showed relatively higher enrichment scores, whereas signatures related to Tregs and NK cells showed relatively lower scores—a pattern consistent with a profibrotic inflammatory milieu. Notably, ADRB2 encodes the β_2_-adrenergic receptor, a Gs-coupled receptor that activates cAMP/PKA signaling, a pathway known to modulate autophagic flux and T-cell effector and regulatory functions in chronic inflammation (28). In this context, the positive correlation between ADRB2 expression and effector CD8^+^ T cell–related signatures, together with its negative association with activated CD4^+^ T cell– and Treg-related signatures, suggests that ADRB2-related autophagy and cAMP signaling may influence the balance between cytotoxic and regulatory T-cell–associated transcriptional programs within keloid lesions. PLK2 is a serine/threonine kinase that regulates selective autophagy and inflammatory signaling; experimental studies have shown that PLK2 modulates macroautophagy and the turnover of aggregation-prone proteins, and can promote immune cell activation via NF-κB–dependent pathways (26). In our dataset, PLK2 expression was negatively correlated with plasmacytoid dendritic cell–related signatures, which have been implicated in collagen deposition through type I interferon and chemokine signaling in fibrotic disorders such as systemic sclerosis (29). Taken together, these findings are consistent with a model in which ADRB2 and PLK2 may act as regulators of autophagy and modulators of immune-related transcriptional patterns, providing a plausible hypothesis for how dysregulated autophagy and immune signaling could jointly contribute to the immunopathogenesis of keloid formation—a hypothesis that will require further confirmation in dedicated mechanistic and immunological studies.

At the histological and protein levels, H&E and Masson’s trichrome staining showed classical fibrotic features in keloid tissues, including dermal thickening and aberrant collagen deposition. Complementary immunohistochemistry and Western blotting indicated higher expression of ADRB2 and PLK2 in keloid samples compared with adjacent normal skin, in line with the transcriptomic findings. These results are compatible with a potential functional association between these genes and keloid pathogenesis, although they should be interpreted as preliminary because of the limited sample size. This integrative research model—combining bioinformatic screening, tissue-level observations, and hypothesis-generating mechanistic inference—illustrates a feasible and translationally oriented framework for biomarker discovery.

KFs exhibited simultaneous upregulation of fibrotic markers (COL1, COL3), autophagy-related proteins (LC3-II, p62), and regulatory molecules ADRB2 and PLK2, indicating that dysregulated autophagy contributes to the persistent fibrotic phenotype. This observation is consistent with previous reports showing that aberrant autophagy promotes fibroblast activation and extracellular matrix accumulation in fibrotic diseases such as systemic sclerosis and intestinal fibrosis (30, 31). Modulation of autophagy revealed that ADRB2 expression decreased following EBSS-induced activation and increased after chloroquine-mediated inhibition, suggesting that ADRB2 expression dynamically responds to autophagic flux and acts downstream of autophagy signaling. This finding aligns with previous evidence showing that β_2_-adrenergic receptor activity is influenced by metabolic stress and autophagy-related regulatory pathways (24, 32). In contrast, PLK2 levels remained stable under both autophagy activation and inhibition, implying that PLK2 functions upstream of autophagic initiation. Given its known regulatory effects on the mTOR and AMPK signaling pathways, PLK2 may influence autophagy through modulation of these upstream nodes (33). Together, these results support a hierarchical regulatory model in which PLK2 acts as an upstream modulator of autophagy, while ADRB2 serves as a downstream effector that reflects the autophagic state in keloid fibroblasts.

By leveraging integration of transcriptomic data and targeted protein-level validation, along with algorithmic cross-validation, this study prioritized ADRB2 and PLK2 as autophagy- and immunity-related candidate genes potentially involved in keloid disease. Furthermore, we outline a preliminary mechanistic hypothesis in which these genes may act as molecular bridges linking autophagy regulation and immune modulation. Future investigations should experimentally dissect the upstream and downstream signaling pathways of ADRB2 and PLK2, clarify their interactions with specific fibroblast subpopulations, and evaluate their suitability as potential therapeutic research targets to inhibit scar formation. Such efforts may ultimately contribute to the development of more precise strategies for the clinical management of keloids.

This study has important limitations. First, histological and protein-level validation was performed in only four paired keloid/normal skin samples, limiting statistical power and generalizability. Second, no functional experiments were conducted to directly assess the causal roles of ADRB2 and PLK2 in autophagy, fibroblast behavior, or fibrosis. Third, immune infiltration analyses relied solely on ssGSEA of bulk transcriptomic data, which reflects gene-set enrichment rather than true immune cell abundance or function. Fourth, the diagnostic model may be affected by overfitting due to the relatively small external validation sets. Therefore, all mechanistic interpretations should be considered preliminary and hypothesis-generating until validated in larger cohorts and functional studies.

## 5 Conclusion

By integrating transcriptomic data, WGCNA, and multiple machine learning algorithms, this study prioritized two autophagy-related hub gene candidates—ADRB2 and PLK2—that may act as potential regulators in keloid pathogenesis. Functional annotation and immune-related analyses suggested associations between these genes and pathways related to fibroblast autophagy, proliferation, and remodeling of the immune microenvironment. Histological and protein-level assays indicated higher expression of ADRB2 and PLK2 in keloid tissues compared with adjacent normal skin, in concordance with the bioinformatic predictions. In fibroblasts, keloid-derived fibroblasts displayed concomitant upregulation of fibrotic markers (COL1/COL3) and autophagy-related proteins (increased LC3-II/LC3-I and p62) together with elevated ADRB2 and PLK2 at baseline; moreover, autophagy modulation in keloid fibroblasts altered ADRB2 expression (decreased after EBSS and increased after chloroquine), whereas PLK2 remained relatively stable. Collectively, these preliminary findings support ADRB2 and PLK2 as autophagy- and immunity-related candidate biomarkers in keloids and raise the possibility that they could serve as potential therapeutic research targets, although their mechanistic roles and clinical applicability require further experimental validation.

## Data Availability

The original contributions presented in the study are included in the article/**Supplementary Material**. Further inquiries can be directed to the corresponding authors.
