# Supplementary figures and images for: Identification and histological validation of autophagy-related core genes ADRB2 and PLK2 in keloids, with integrated immune infiltration analysis
Source: Front Immunol. 2026 Feb 9;17:1724230. doi: 10.3389/fimmu.2026.1724230 (PMC12926160; doi:10.3389/fimmu.2026.1724230)

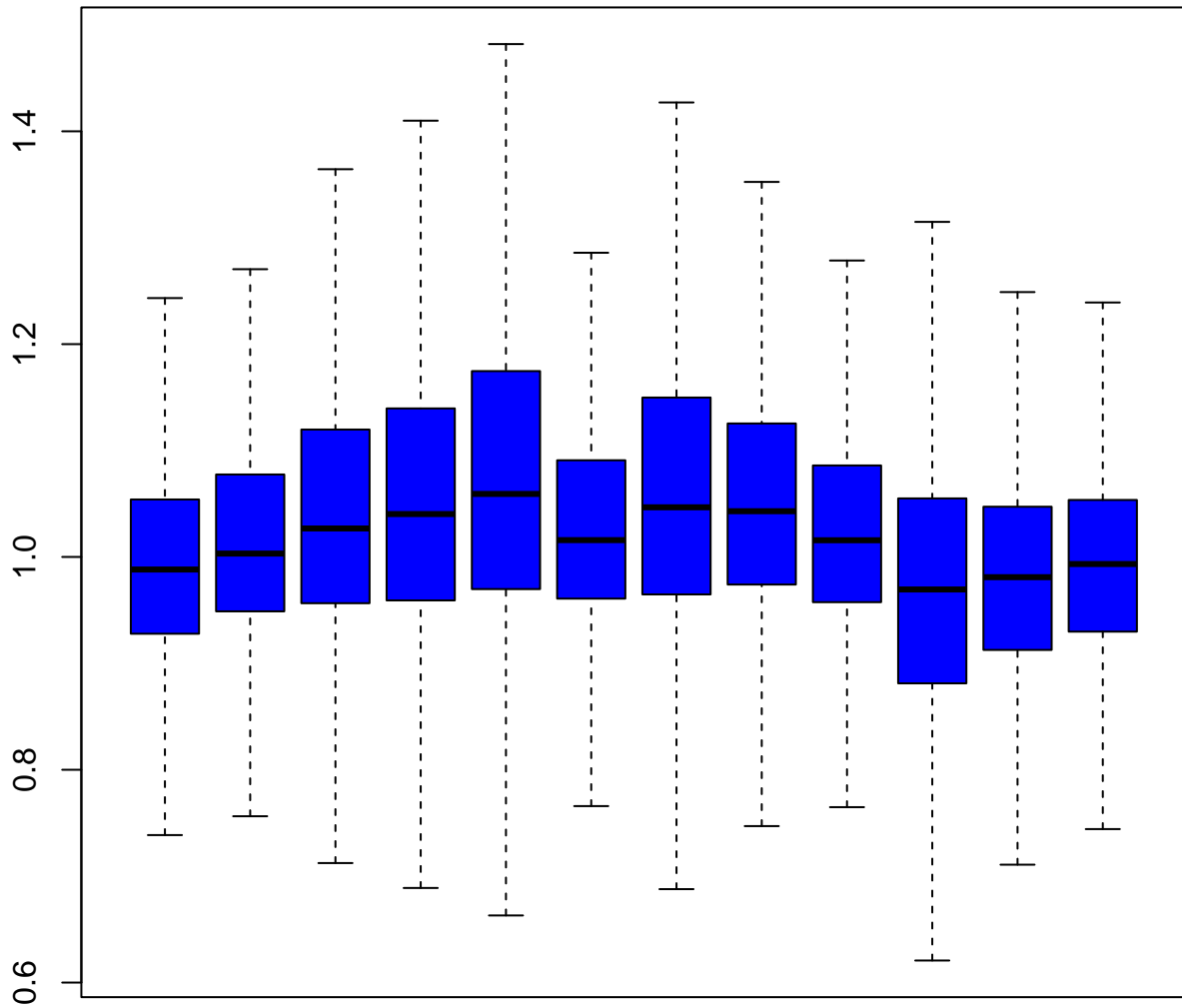

Supplement: Supplementary file 1 [file DataSheet1.pdf]

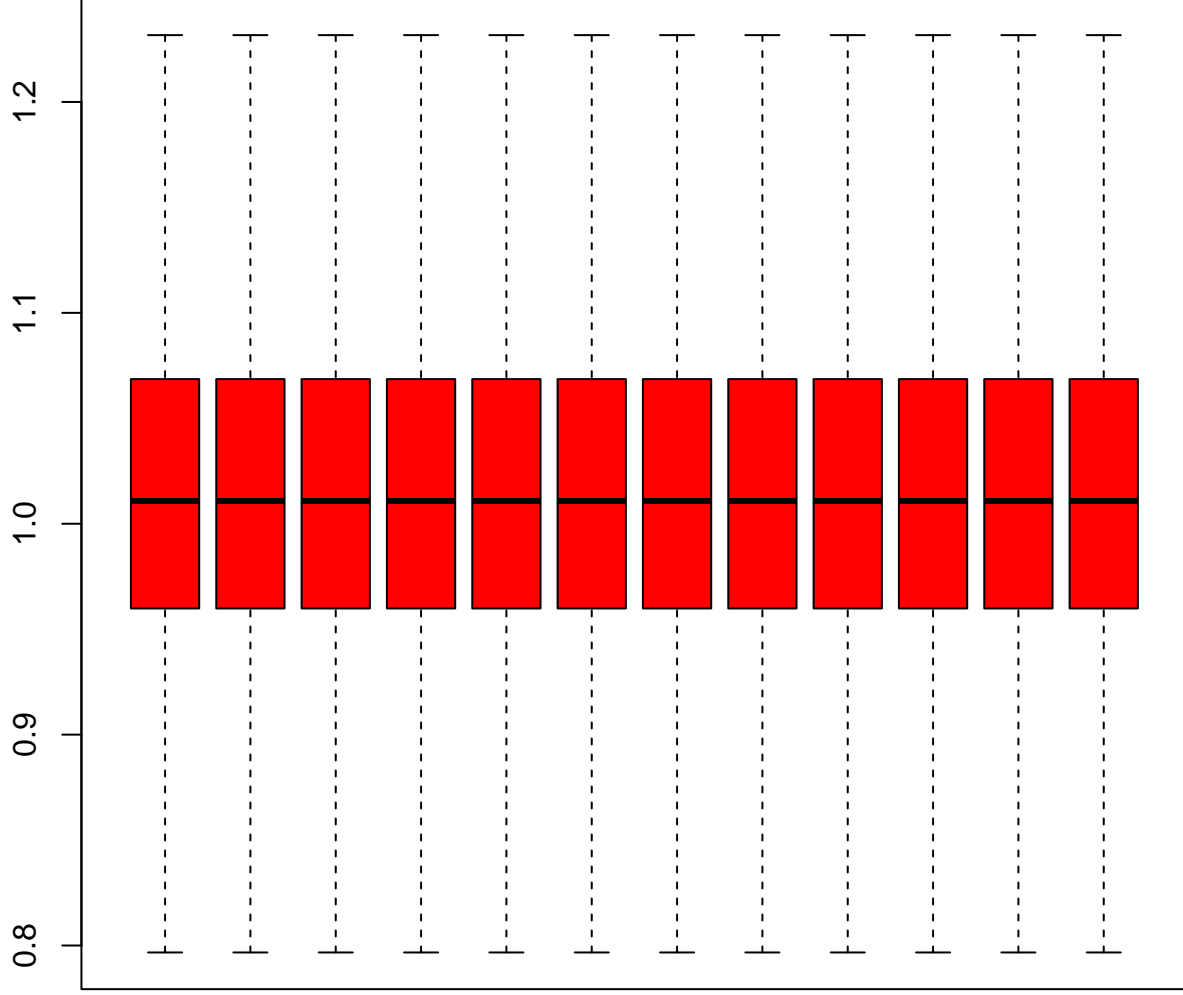

Supplement: Supplementary file 2 [file DataSheet2.pdf]

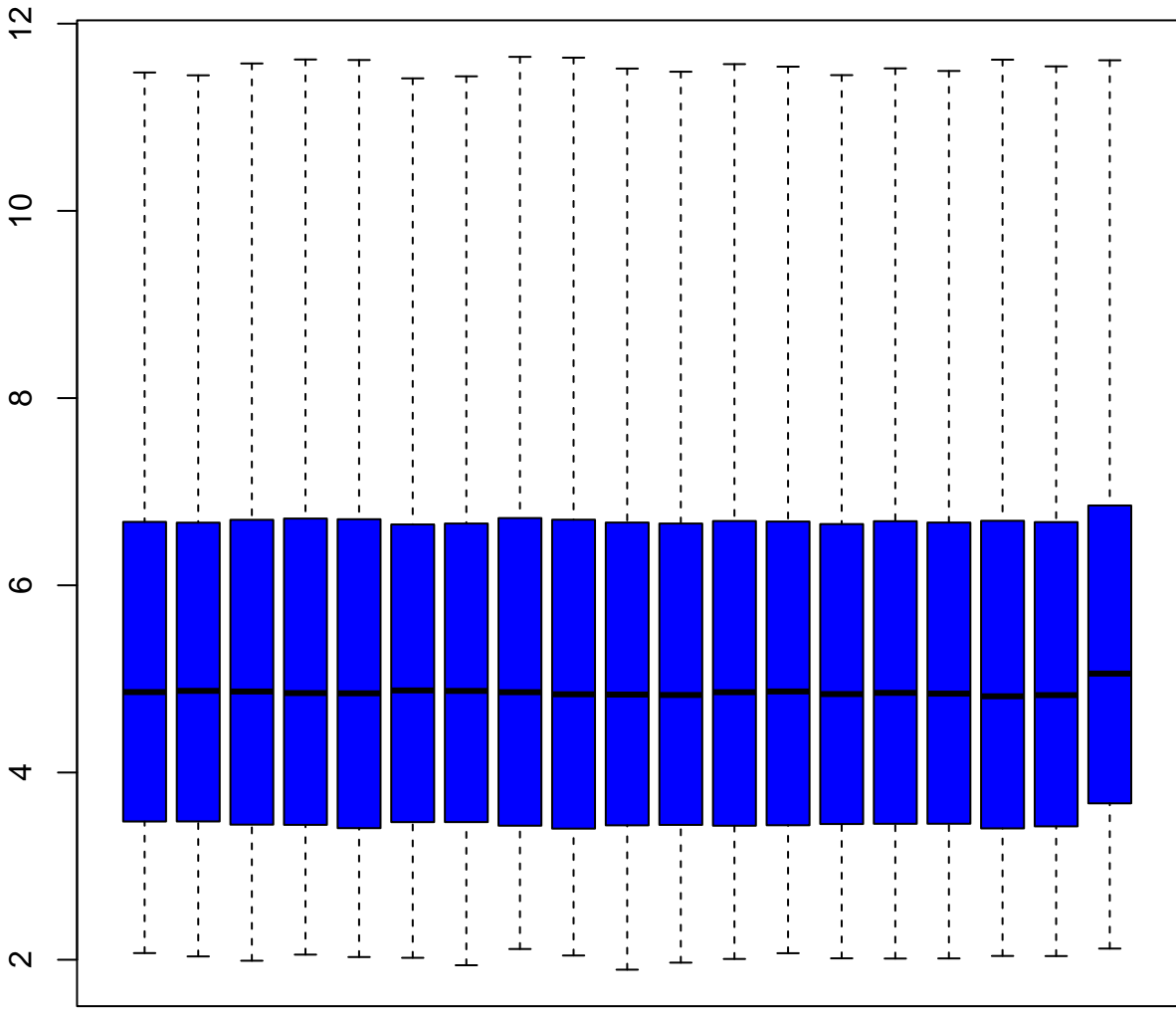

Supplement: Supplementary file 3 [file DataSheet3.pdf]

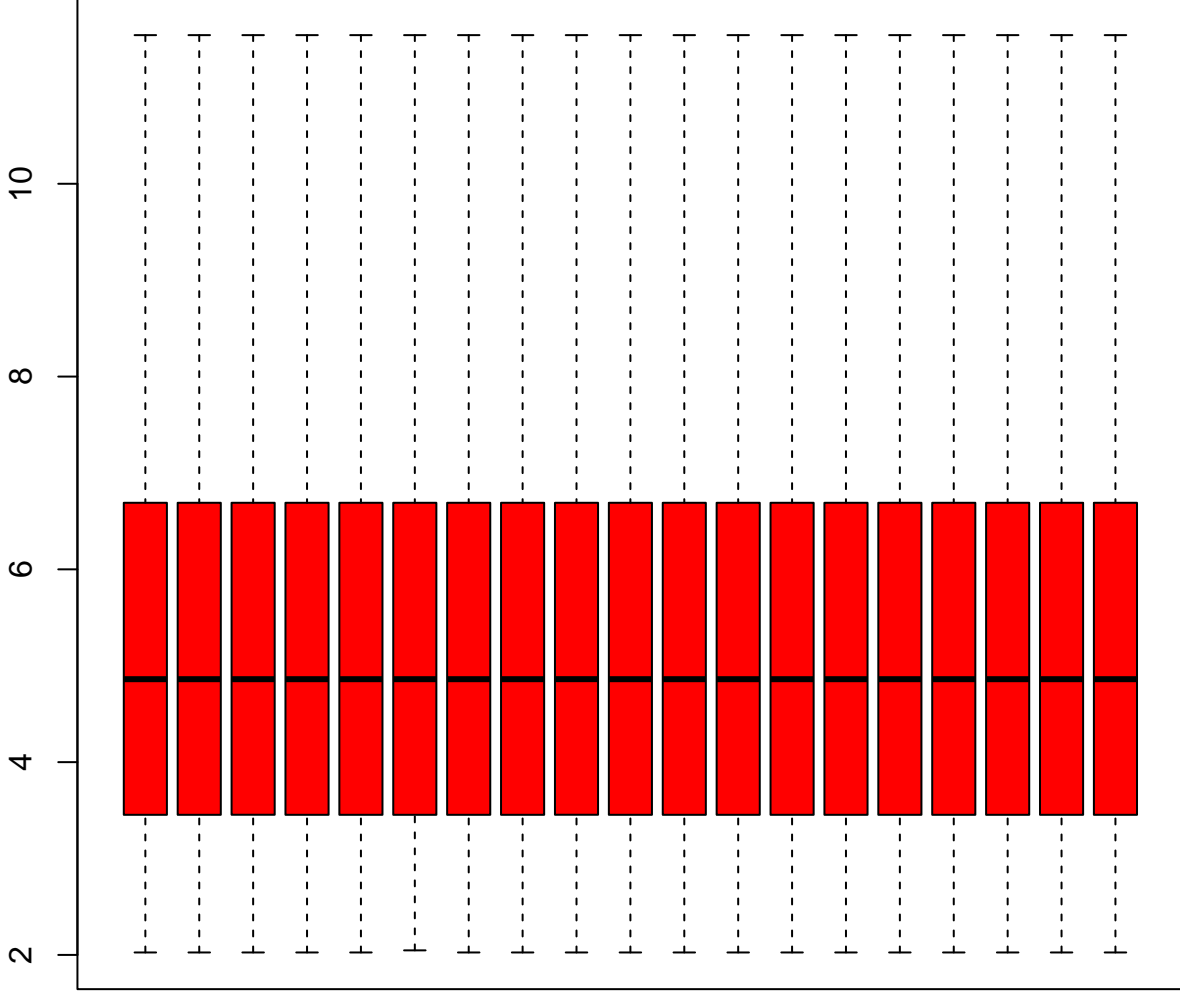

Supplement: Supplementary file 4 [file DataSheet4.pdf]

## Before batch correction

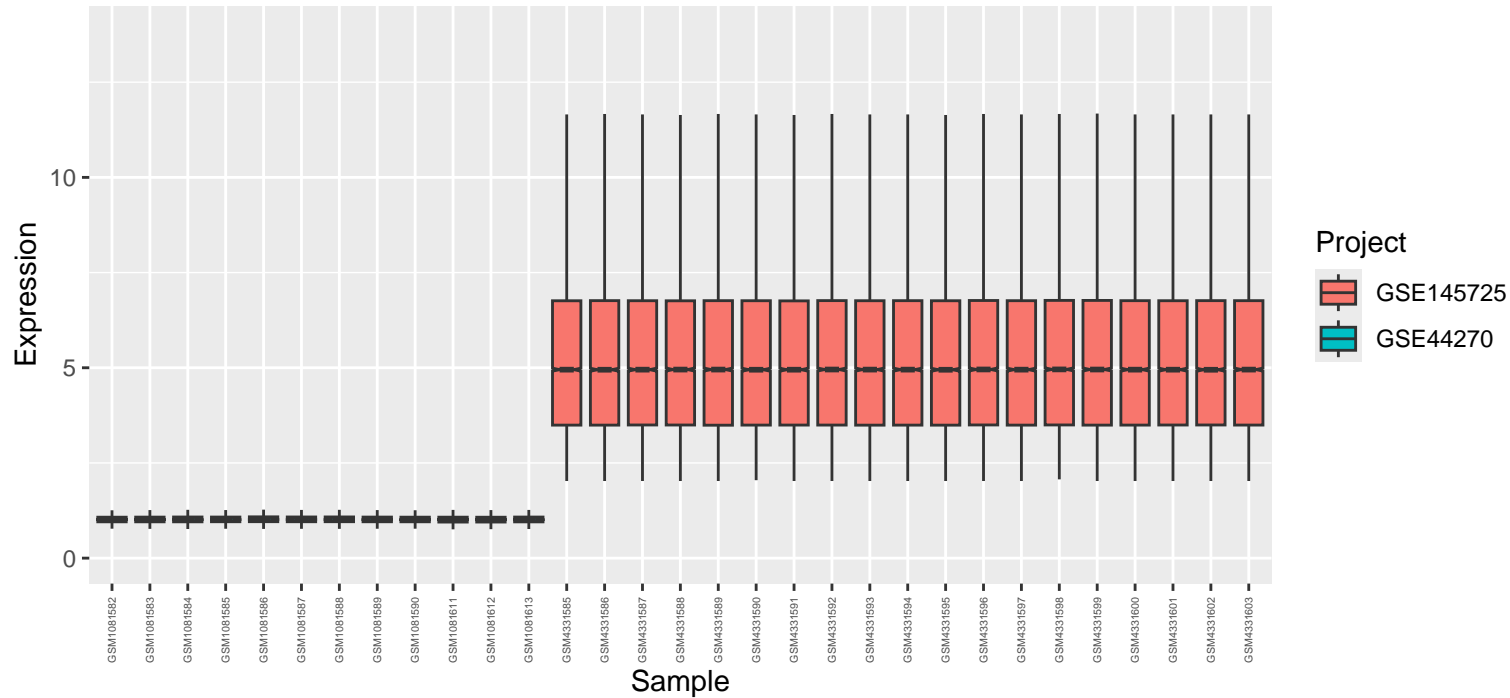

Supplement: Supplementary file 5 [file DataSheet5.pdf]

# After batch correction

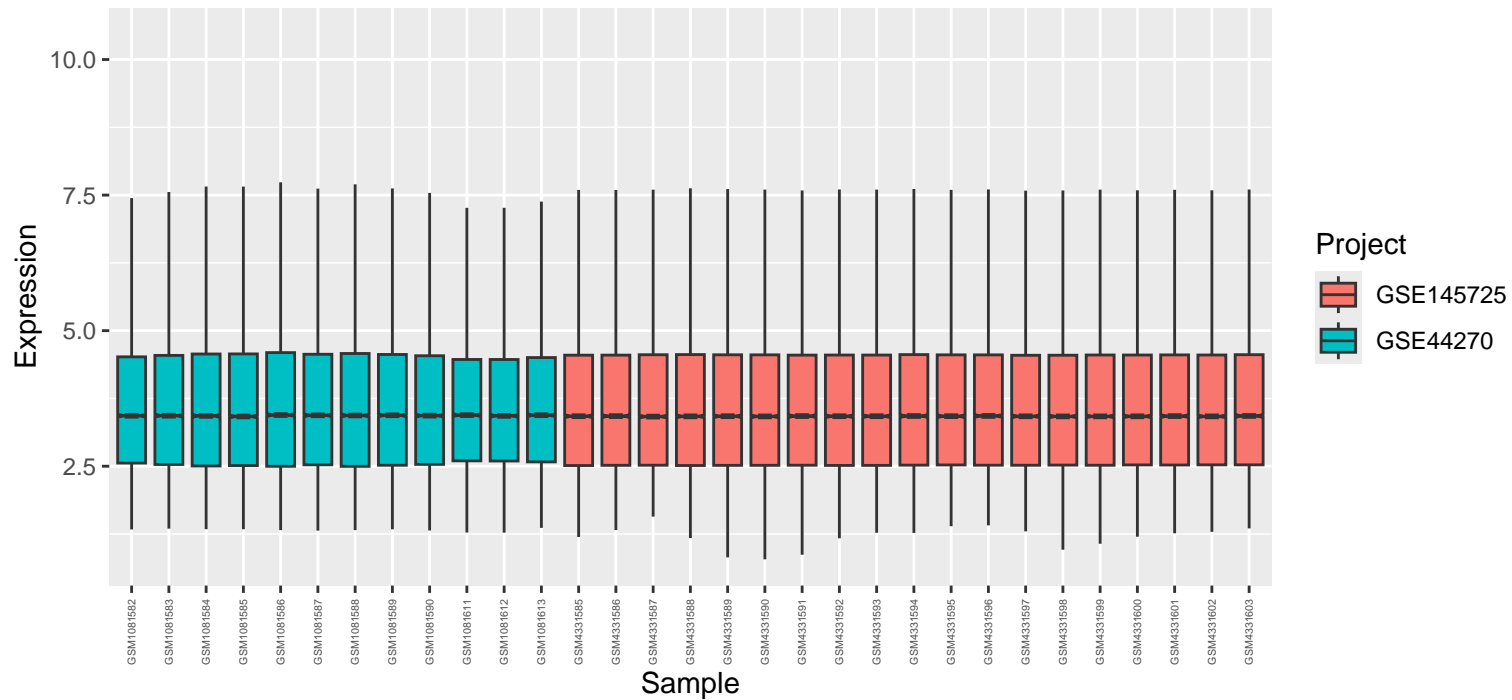

Supplement: Supplementary file 6 [file DataSheet6.pdf]

Sample dendrogram and trait heatmap

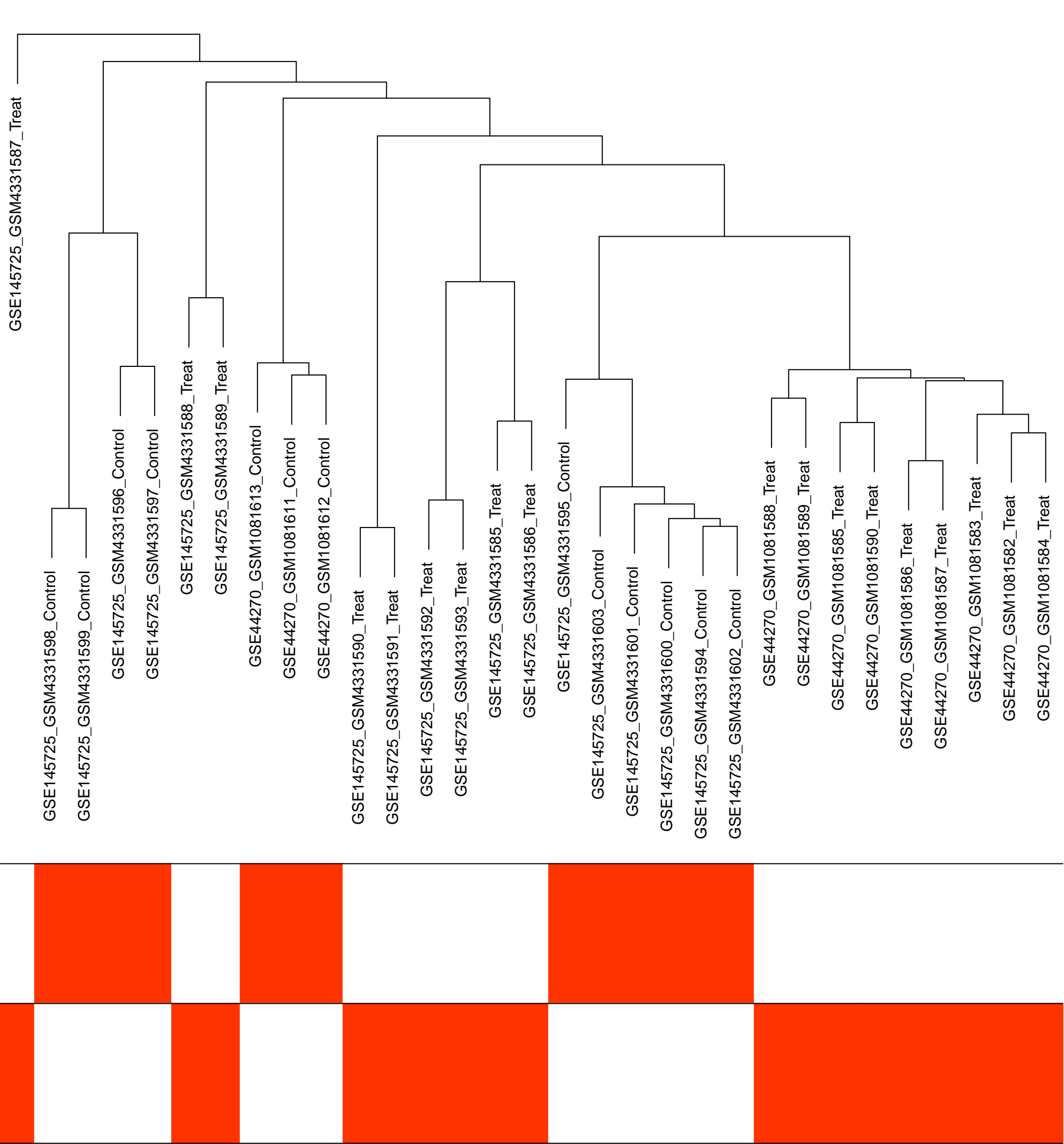

Supplement: Supplementary file 7 [file DataSheet7.pdf]

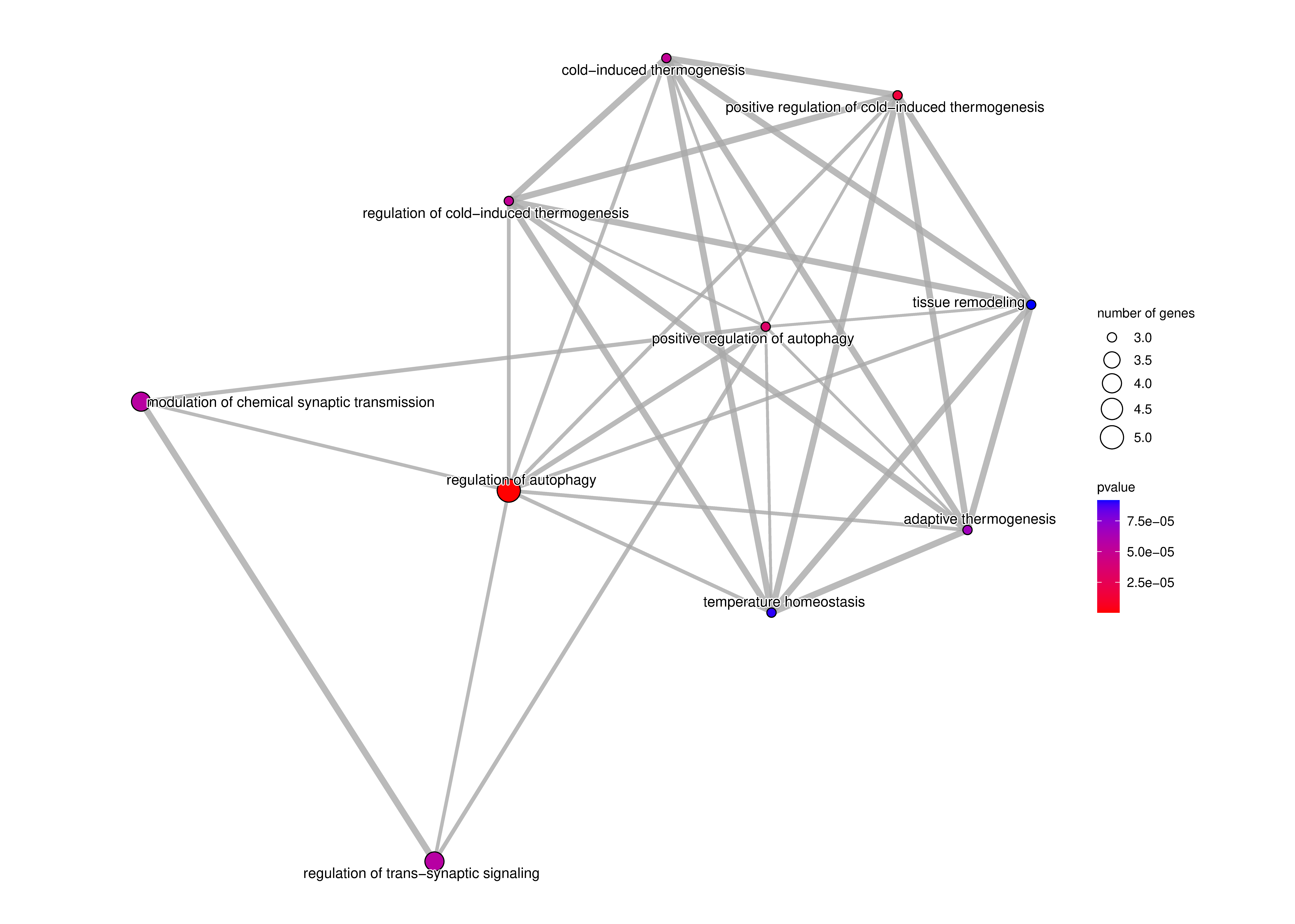

Supplement: Supplementary file 10 [file Image1.tiff]

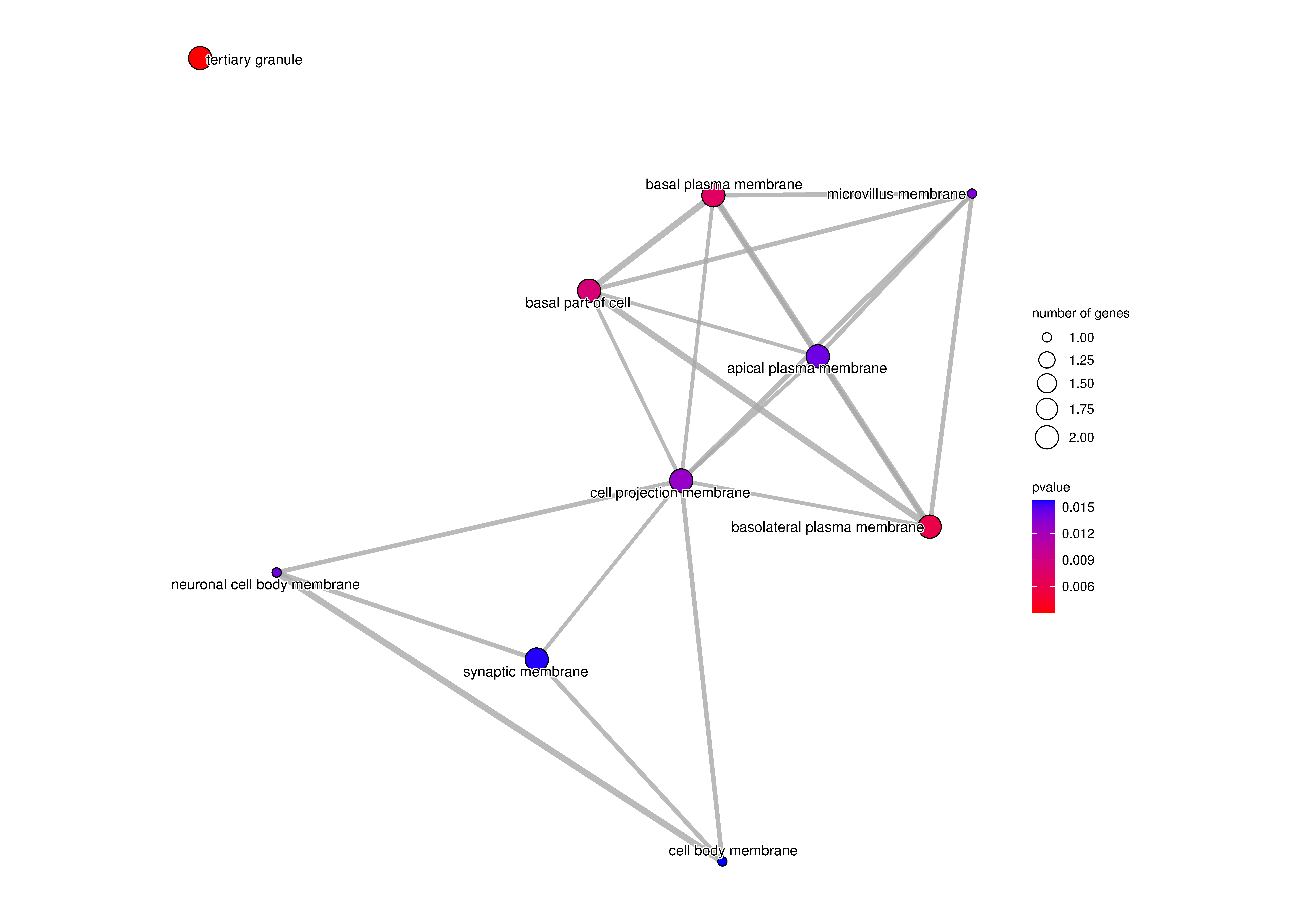

Supplement: Supplementary file 11 [file Image2.tiff]

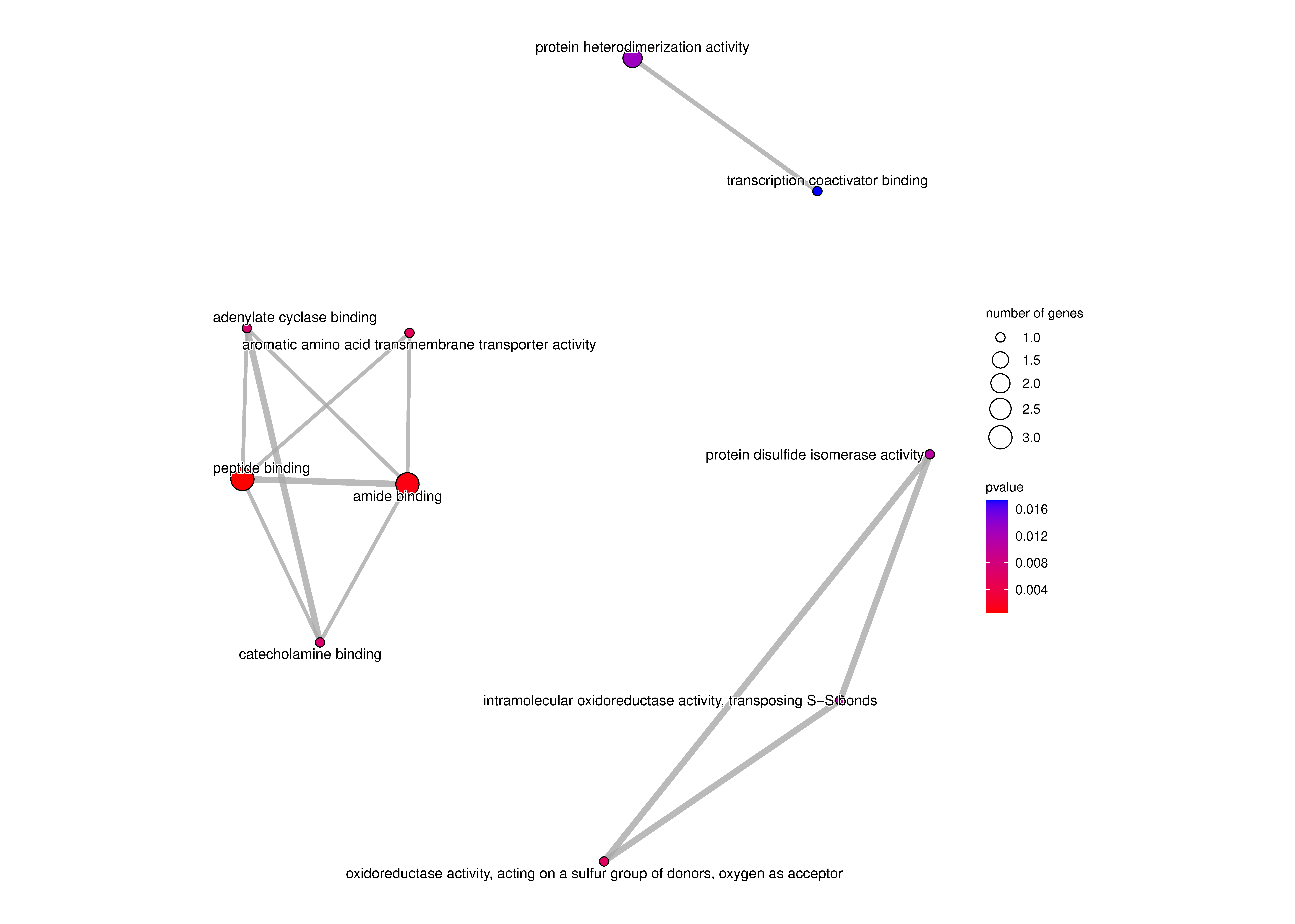

Supplement: Supplementary file 12 [file Image3.tiff]

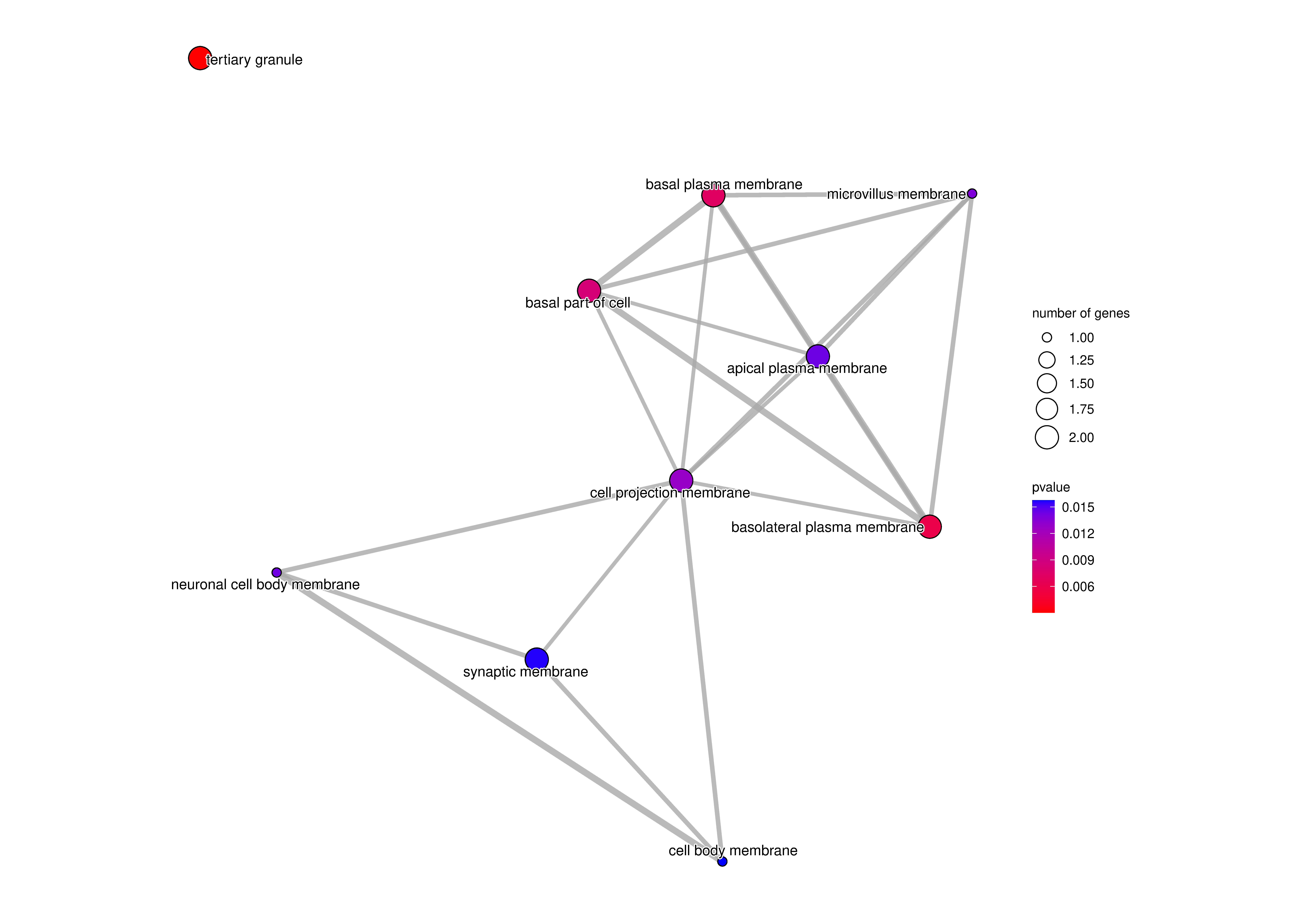

Supplement: Supplementary file 14 [file Image5.tiff]

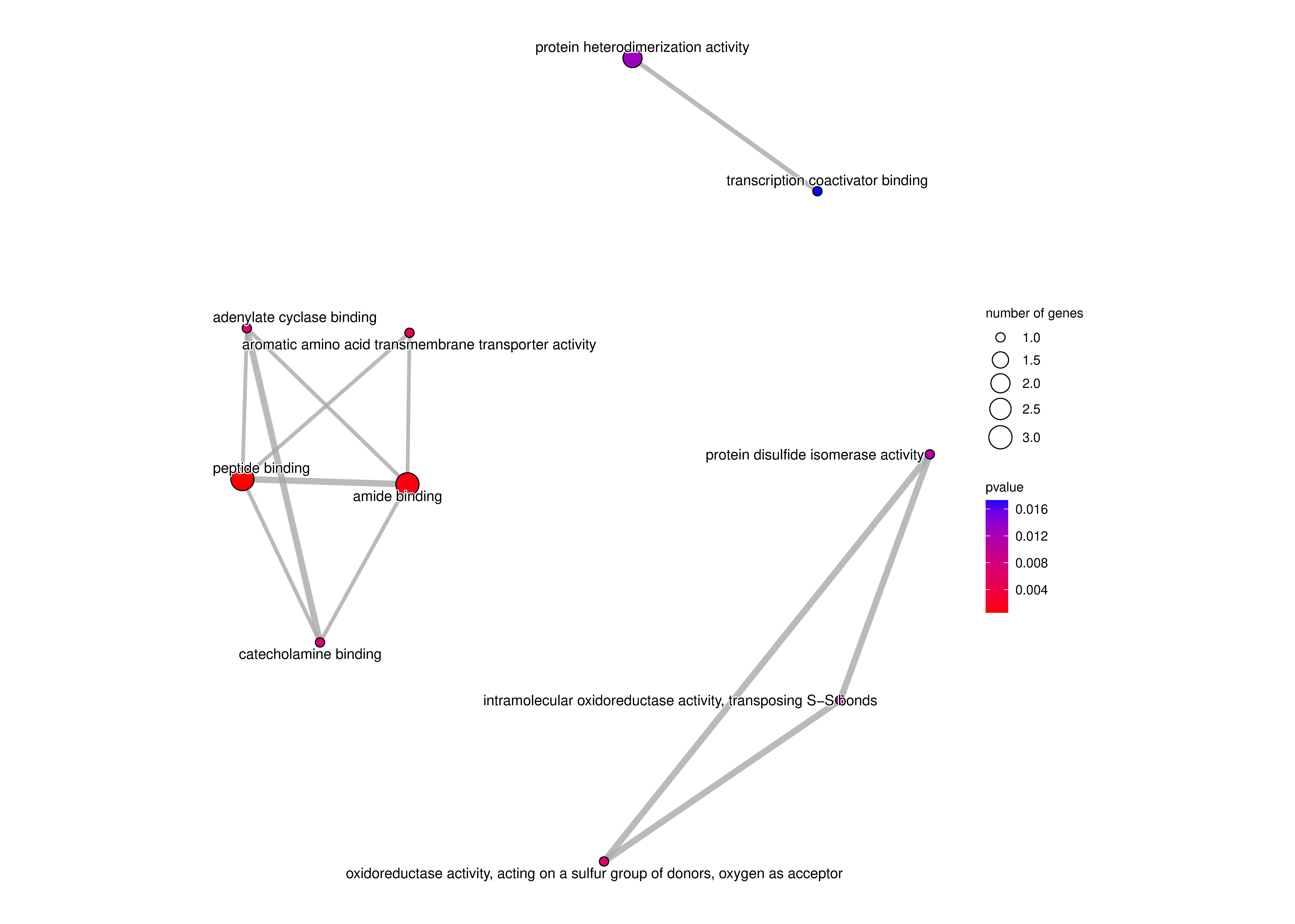

Supplement: Supplementary file 15 [file Image6.tiff]
